# Impact of Deleterious Mutations on Structure, Function and Stability of Serum/Glucocorticoid Regulated Kinase 1: A Gene to Diseases Correlation

**DOI:** 10.3389/fmolb.2021.780284

**Published:** 2021-11-03

**Authors:** Mohamed F. AlAjmi, Shama Khan, Arunabh Choudhury, Taj Mohammad, Saba Noor, Afzal Hussain, Wenying Lu, Mathew Suji Eapen, Vrushali Chimankar, Philip M Hansbro, Sukhwinder Singh Sohal, Abdelbaset Mohamed Elasbali, Md. Imtaiyaz Hassan

**Affiliations:** ^1^ Department of Pharmacognosy, College of Pharmacy, King Saud University, Riyadh, Saudi Arabia; ^2^ Drug Discovery and Development Centre (H3D), University of Cape Town, Cape Town, South Africa; ^3^ Department of Computer Science, Jamia Millia Islamia, New Delhi, India; ^4^ Centre for Interdisciplinary Research in Basic Sciences, Jamia Millia Islamia, New Delhi, India; ^5^ Respiratory Translational Research Group, Department of Laboratory Medicine, School of Health Sciences, College of Health and Medicine, University of Tasmania, Launceston, TAS, Australia; ^6^ Centre for Inflammation, Centenary Institute and University of Technology Sydney, School of Life Sciences, Faculty of Science, Sydney, NSW, Australia; ^7^ Priority Research Centre for Healthy Lungs and Hunter Medical Research Institute, The University of Newcastle, Newcastle, NSW, Australia; ^8^ Clinical Laboratory Science, College of Applied Medical Sciences-Qurayyat, Jouf University, Sakakah, Saudi Arabia

**Keywords:** serum/glucocorticoid regulated kinase 1, deleterious mutations, single amino acid substitutions, molecular dynamics simulation, essential dynamics

## Abstract

Serum and glucocorticoid-regulated kinase 1 (SGK1) is a Ser/Thr protein kinase involved in regulating cell survival, growth, proliferation, and migration. Its elevated expression and dysfunction are reported in breast, prostate, hepatocellular, lung adenoma, and renal carcinomas. We have analyzed the SGK1 mutations to explore their impact at the sequence and structure level by utilizing state-of-the-art computational approaches. Several pathogenic and destabilizing mutations were identified based on their impact on SGK1 and analyzed in detail. Three amino acid substitutions, K127M, T256A, and Y298A, in the kinase domain of SGK1 were identified and incorporated structurally into original coordinates of SGK1 to explore their time evolution impact using all-atom molecular dynamic (MD) simulations for 200 ns. MD results indicate substantial conformational alterations in SGK1, thus its functional loss, particularly upon T256A mutation. This study provides meaningful insights into SGK1 dysfunction upon mutation, leading to disease progression, including cancer, and neurodegeneration.

## Introduction

Cancer progression is the result of malfunction at multiple cellular levels, including abnormal gene expression, metabolic conditions, abnormal signal transduction, epithelial to mesenchymal transition, genetic, and epigenetic alterations (Sekido, 2010; Mahmood et al., 2017; Lu et al., 2020). Alterations at genomic and proteomic levels cause significant changes to protein structure and function, resulting in the onset and progression of many complex diseases, such as cancer and neurodegeneration (Baak et al., 2003). Serum/glucocorticoid regulated kinase 1 (SGK1) is a member of the AGC family of Serine/Threonine protein kinases that regulate the survivability and growth of cells (Lang et al., 2010). It is involved in regulating cell cycle progression, proliferation, differentiation and apoptosis, and is associated with the onset and progression of various cancers in humans (Sang et al., 2021). Its elevated expression and dysfunction are linked with multiple pathological conditions, including hypertension, ischemia, diabetic neuropathy, trauma, and neurodegenerative diseases (Eapen et al., 2019). SGK1 is acutely regulated at various levels, including gene transcription and post-translationally by phosphorylation and ubiquitination. It is expressed in several tissues, including the spleen, thymus, bone marrow, breast, prostate, and oral epithelial (Eapen et al., 2019).

SGK1 remains under strict transcriptional control even with various external stimuli such as cell stress and hormones, such as glucocorticoids and mineralocorticoids (O’Keeffe et al., 2013). It is encoded by the *SGK1* gene localized on chromosome 6 in the region 6q23 consisting of 148,867 bases with 14 coding exons (Waldegger et al., 1998). The protein comprises 431 amino acids with a molecular mass of ∼49 kDa (Zhao et al., 2007). The active site (proton acceptor) and ATP binding site of SGK1 are located at Asp222 and Lys127, respectively, (Zhao et al., 2007). Most of the SGK1 structure has a common kinase fold, but the structure near its active site is unique compared to other kinases, and the main difference is near the ATP binding site (Zhao et al., 2007). This is crucial for its functional activity, and any structural alteration at the ATP binding site can cause SGK1 dysfunction, which may lead to disease progression.

A single amino acid substitution or naturally occurring mutations are associated with several complex diseases, including cancers. Deleterious mutations at the genomic and/or proteomic level have significant impacts on human health. These mutations in SGK1, especially near its active site region, especially at ATP binding site, cause significant structural alterations and its dysfunction, which may promote disease progression (Snyder et al., 2002; Boehmer et al., 2003; Henke et al., 2004). There are numerous reports of several naturally occurring mutations in SGK1, but their roles in pathogenesis at the structural level have not been widely studied (Kobayashi and Cohen, 1999; Snyder et al., 2002). Biophysics-based computational methods are valuable in studying the impact of mutations on protein structure and function, and there is intense current interest in such studies (Amir et al., 2019a; Amir et al., 2019b; Choudhury et al., 2021; Habib et al., 2021).

Several methods have been developed to identify deleterious or disease-causing mutations within human protein sequences. These methods predict the deleteriousness of an amino acid substitution on the basis of physicochemical properties, structure, and cross-species conservation analysis (Ng and Henikoff, 2006; Chun and Fay, 2009). Identification of deleterious mutations in an individual has the potential to influence both the prevention and personalized interventions in disease.

Here, we performed an in-depth analysis of genomic and proteomic alterations in SGK1 using state-of-the-art computational approaches (Choudhury et al., 2021; Habib et al., 2021; Umair et al., 2021). We examined a range of mutations and characterized their deleterious impact on the structure and function of SGK1, which may contribute to disease development and progression, such as cancer and neurodegeneration.

## Materials and Methods

### Retrieval of Data

The FASTA sequence of SGK1 was taken from the UniProt (UniProt ID: O00141). A list of mutations was taken from the dbSNP (Sherry et al., 2001) and Ensembl (Hubbard et al., 2002) databases and an extensive literature survey. Data redundancy, including duplicate variants, was removed during preprocessing. The structural coordinates of human SGK1 were retrieved from the RCSB Protein Data Bank (PDB), using the PDB identifier 2R5T (Berman et al., 2000).

### Sequence-Based Prediction

#### PolyPhen2

PolyPhen-2 is a sequence-based mutation analysis tool, and it takes the FASTA sequence as input (Ramensky et al., 2002). Through conservative and physical properties, this tool calculates the potentially deleterious effects of a mutation. It incorporates multiple sequence alignments, a machine learning-based classifier, and optimized for high-throughput NGS data analysis. It provides the Position-Specific Independent Count (PSIC) score for the mutant protein and estimates the score difference with the native protein. If the PSIC score is higher than 0.09, then the amino acid substitution is considered deleterious. PolyPhen-2 is accessible through http://genetics.bwh.harvard.edu/pph2/(Adzhubei et al., 2010).

#### PROVEAN

PROVEAN estimates the impact of mutations on the protein’s functionality based on the delta alignment score (Choi and Chan, 2015). For a deleterious mutation, the PROVEAN score is less than −2.5, whereas for neutral non-synonymous mutations, scores are greater than −2.5. The PROVEAN web server comprises three tools, PROVEAN Protein, PROVEAN Protein Batch, and PROVEAN Genome Variants. The PROVEAN Protein Batch tool also returns the result of SIFT tool and can process a large number of protein variants. The input for this function takes amino acid substitutions and supports public protein identifiers such as NCBI RefSeq, UniProt, and Ensembl. PROVEAN is accessible through http://provean.jcvi.org/.

#### SIFT

The SIFT tool considers sequence homology and physical properties of amino acid residues to determine whether the mutation is deleterious or not. It also depends on the evolutionary conservation of amino acids in protein families. The highly conserved amino acids tend to be intolerant to substitutions, and most of the less conserved ones tolerate the substitutions. (Kumar et al., 2009). The SIFT score for a non-tolerable mutation is less than or equal to 0.05 (Ng and Henikoff, 2003; Kumar et al., 2009). SIFT is accessible through http://sift.jcvi.org/.

#### FATHMM

FATHMM is another web-based application for predicting the functional impact of mutations on proteins (Shihab et al., 2013). The coding variants can be analyzed for inherited diseases, such as cancer and complex diseases. FATHMM comprises two algorithms: weighted and unweighted, of which we used the unweighted algorithm for predicting the ontology of inherited diseases. The unweighted method searches conserved residues through an approach based on fundamental amino acid probabilities. The weighted method assigns pathogenicity weights that correlate with disease-causing amino acids, with sequence conservation found through searching Hidden Markov models (HMMs). FATHMM is accessible through http://hathmm.biocompute.org.uk.

### Structure-Based Prediction

#### mCSM

mCSM is a web-based predictor that uses a graph-based approach to predict the impact of missense mutations on protein stability (Pires et al., 2014). The predictive models in mCSM are trained with the atomic distance patterns of different amino acid residues. mCSM covers a wide range of proteins for disease association of mutations. The calculated mCSM score (ΔΔ*G*) for a destabilizing mutation is less than 0. mCSM is accessible through http://biosig.unimelb.edu.au/mcsm/.

#### SDM

SDM is a webserver that calculates the change in protein stability upon mutation. The protein stability change for a mutation is calculated using PDB coordinate files and environment-specific amino acid substitution tables (Overington et al., 1992; Pandurangan et al., 2017). If the ΔΔ*G* is higher than 0 for a mutation, SDM predicts it as a destabilizing mutation. SDM is accessible through http://marid.bioc.cam.ac.uk/sdm2.

#### MAESTROweb

MAESTROweb is a stability prediction tool that takes a multi-agent approach to estimate the free energy difference between the native and mutant protein. It accepts PDB coordinates as input and uses a machine learning-based approach to calculate the change in the Gibbs free energy value. If the MAESTRO score is less than 0 for a mutation, then it predicts that the mutation is destabilizing (Laimer et al., 2015). MAESTROweb is accessible through https://pbwww.che.sbg.ac.at/maestro/web.

#### PremPS

PremPS evaluates the effects of mutations on protein stability by estimating the quantitative change in unfolding Gibbs free energy (Chen et al., 2020). Predictions are based on the protein structure. The PremPS tool uses a random forest (RF) regression scoring function. The tool was trained with experimental data of unfolding Gibbs free energy changes (ΔΔG) for 5,296 mutations from 131 proteins. To improve the performance of the tool and the datasets, reverse mutations are also incorporated. For the forward mutations (ΔΔG_wt→mut_), three-dimensional structures of native proteins were taken from the PDB. The BuildModel module of FoldX is used for reverse mutations (ΔΔG_mut→wt_). The PremPS energy function is based on 10 evolutionary and structure-based features which belong to six categories. PremPS is accessible through https://lilab.jysw.suda.edu.cn/research/PremPS/.

### Disease Phenotype Prediction

#### SNPs and GO

SNPs and GO is an SVM-based webserver that identifies pathogenic non-synonymous substitutions (Capriotti et al., 2013). It uses gene ontology (GO) annotations to classify a missense variant into a disease-related or neutral variant. It requires amino acid sequence/SwissProt code, GO terms, and amino acid substitutions as input. An SNPs and GO score of more than 0.5 indicates a disease-causing mutation, and this tool also gives the result of PANTHER and PhD-SNP. SNPs and GO is accessible through https://snps.biofold.org/snps-and-go/snps-and-go.html.

#### PON-P2

PON-P2 is a machine learning-based web tool for analyzing mutations in human proteins (Niroula et al., 2015). It divides the non-synonymous substitutions into pathogenic, neutral and unknown classes. It can proficiently and rapidly analyze large-scale variant datasets. For identifier submission, it takes mutation and one of Ensembl or Entrez, UniProtKB identifiers. PON-P2 uses evolutionary sequence conservation and physical and biochemical properties of a protein to calculate the potential pathogenicity of mutations. GO annotations and functional annotations are also used based on their availability. PON-P2 is accessible through http://structure.bmc.lu.se/PON-P2/.

#### PMut

PMut is one of the webservers for disease phenotype identification. PMut consists of a network-based classifier, and datasets are obtained from the manually created Swiss-Prot database. Physiochemical properties and sequence conservation are two of the main features of the tool. If the PMut score for a mutation is greater than 0.5, the mutation is considered pathogenic. The updated version also has the option to generate new predictors for specific protein families. It also has a database of the pre-estimated predictions (López-Ferrando et al., 2017). PMut is accessible through http://mmb.irbbarcelona.org/PMut.

### Analysis of Conserved Residues

ConSurf is a webserver for determining the degree of conservation of amino acids in a specific position using multiple sequence alignment (Ashkenazy et al., 2016). The evolutionary conservation of residues is critical to understand the function and structure of a protein. The ConSurf score extends from 1 to 9, where 1 signifies the least conserved residue, and 9 is for highly conserved residues. ConSurf is accessible through https://consurf.tau.ac.il/.

### Analysis of Aggregation Propensity

**FIGURE 1:**
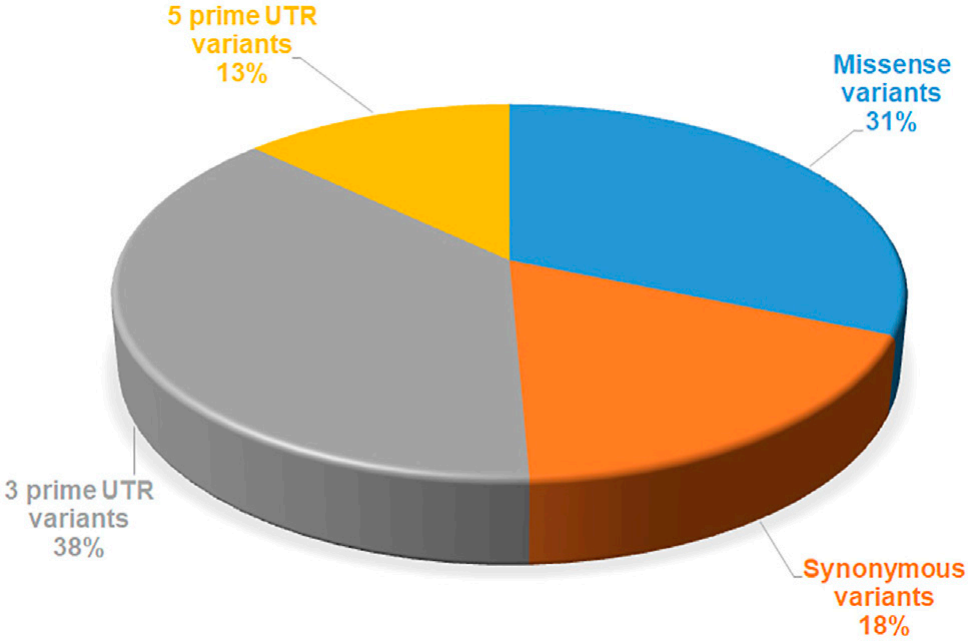
Number of mutations reported in SGK1, extracted from the dbSNP database.

SODA is a web-based application used in studying the aggregation, disorder, helix, and strand propensity that occur due to single nucleotide polymorphisms. It is used to study various mutations, including insertion, deletion, substitution, and duplication in a protein molecule. The SODA score is based on the difference in solubility between the native and mutant protein (Paladin et al., 2017). SODA is accessible through http://protein.bio.unipd.it/soda/. The bioinformatics approach and various applications used are illustrated in Figure 1.

### MD Simulations

#### Systems Preparation and Simulation Protocol

The native structure of SGK1 downloaded from the PDB was processed for deleting crystallographic water and adding missing atoms. The mutant models were prepared by utilizing the mutagenesis wizard of PyMOL (DeLano, 2002). All-atom MD simulation and potential energy minimization were performed on SGK1 and its mutants models using the Amber 18 software. The Amber 18 forcefield FF14SB was applied during the simulation protocol. Energy-minimized structures of all four systems (one wild-type (WT) and three mutants) were taken as the starting coordinates for the simulation. All four structures were solvated in a cubic TIP3P water model. Periodic boundary conditions were set so that the number of particles, pressure, and temperature are constant during the simulation. The simulation setups were neutralized by adding an appropriate number of counterions. The temperature at 300 K was retained by employing the Berendsen algorithm with a coupling time of 0.2. All atoms of the protein systems were placed at a distance of 10 Å from the edges of the cubic box. The minimized simulation setups were then equilibrated for 1,000 ps at 300 K via the position-restrained simulation approach for solvation. The equilibrium setups were then subjected to final MD runs for 200 ns. The Particle mesh Ewald (PME) method was employed for long-range Coulombic interactions. The SHAKE algorithm was used to determine the bond lengths between hydrogen atoms, with a time step of 2 fs (Andersen, 1983).

### Post-Dynamic Trajectory Analysis

The generated trajectories were analyzed using the conventional utilities of the Amber 18 suite to obtain RMSD, RMSF, *R*_g_, SASA, intramolecular hydrogen bonding, secondary structure analysis, distance cross-correlation matrix and principal component analysis (PCA). The structural coordinates of all four systems were collected for every 1 ps, and trajectory curves were computed via the CPPTRAJ module (Roe and Cheatham, 2013) of Amber 18. The number of intramolecular hydrogen bonds was defined based on a donor-hydrogen-acceptor angle >90 nm and a donor-acceptor distance <3.9 nm. VMD (Humphrey et al., 1996) was used for molecular visualization of MD trajectories, and QtGrace was employed to generate plots of MD results.

### Dynamics of the Cross-Correlation Matrix

The dynamics of the cross-correlation matrix (DCCM) were explored to determine coordinate aberrations and behaviors in C_α_ atoms of SGK1 and its mutant models. The i and j cross-correlation factors of C_α_ atoms can be calculated as:

$$\;C_{ij}=\frac{<\text{Δri}.\text{Δrj}>}{{\left(<\Delta r_{i}^{2}><\Delta r_{j}^{2}>\right)}^{\frac{1}{2}}}$$ (1)

where Δr_i,j_ is the movement of i^th^ and j^th^ atom average point and angle braces indicated over the complete curves. Correlated movements are denoted by C_ij_ = 1; however, C_ij_ = −1 is supposed to be highly anti-correlated movements. The divergence of atomic movements from 1 to −1 describes that i and j movements are correlated and anti-correlated.

### PCA

PCA is a valuable approach to explore conformational movements in a protein (David and Jacobs, 2014). PCA models atomic movements of protein conformation by retaining dimensional reduction from simulated trajectories (Naqvi et al., 2018; Amir et al., 2019b; Fatima et al., 2019; Mohammad et al., 2019). We performed PCA through the covariance matrix C, based on the atomic coordinates and their corresponding eigenvalues (Papaleo et al., 2009). The generation of positional covariance matrix C can be explained as:

$$C_{i}=\;<\left(q_{i}-<q_{i}>\right)\left(q_{j}-<q_{j}>\right)>\left(i,j=1,2,\ldots ,3N\right)$$ (2)

where q_i_ and q_j_ represent the Cartesian coordinates for the i^th^, j^th^ position of the C_α_ atom and N is the number of C^α^ atoms.

## Results

**FIGURE 2:**
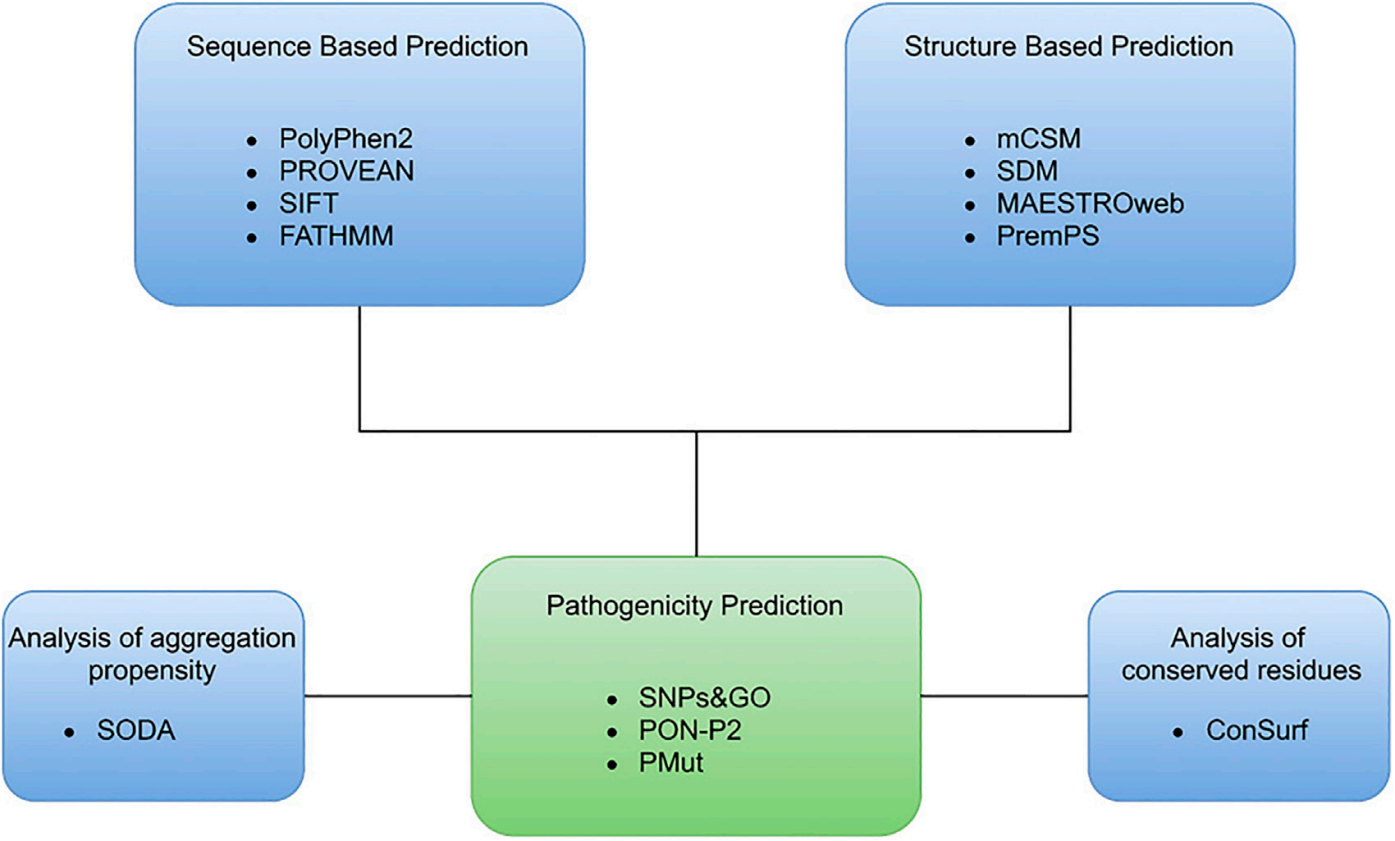
Overview of representation of the computational approach used to identify the deleterious mutations in SGK1.

A set of 156 reported mutations were extracted from the dbSNP and Ensembl databases. PubMed was also used to retrieve mutations through a literature search. The identification of the structural and functional impact of mutations on the SGK1 protein was performed step-by-step. All mutations were analyzed through sequence-based and structure-based methods to define deleterious mutations with high confidence. The sequence-based approach included four web-based tools, PolyPhen2, PROVEAN, SIFT and FATHMM, and the structure-based approach included mCSM, SDM, MAESTROweb, and PremPS. These eight tools separated deleterious/destabilizing mutations from stabilizing/neutral mutations, along with those of unknown significance. Further progression was made by analyzing the pathogenicity of high confidence mutations obtained through the previous two approaches. Pathogenicity of high confidence mutations was predicted through SNPs and GO, PON-P2, and PMut web servers. The distribution of different types of mutations in the SGK1 is depicted in Figure 2.

### Identification of Deleterious Mutations

The analysis includes multiple tools to generate more accurate results by eliminating false Predictions. PolyPhen2, PROVEAN, SIFT, and FATHMM were used as part of the sequence-based approach. The SIFT web tool is based on the physical properties of a protein and separates the mutations into tolerated and intolerant substitutions. A higher tolerance score indicates a lower impact of a mutation on the protein function and vice versa (Ng and Henikoff, 2003).

PolyPhen-2 is another tool based on an iterative greedy algorithm and classifies the mutations into three categories: probably damaging (score >0.96), possibly damaging (score >0.2 and <0.96), and benign (score <0.2). To improve accuracy, two other tools PROVEAN and FATHMM tools were used.

**FIGURE 3:**
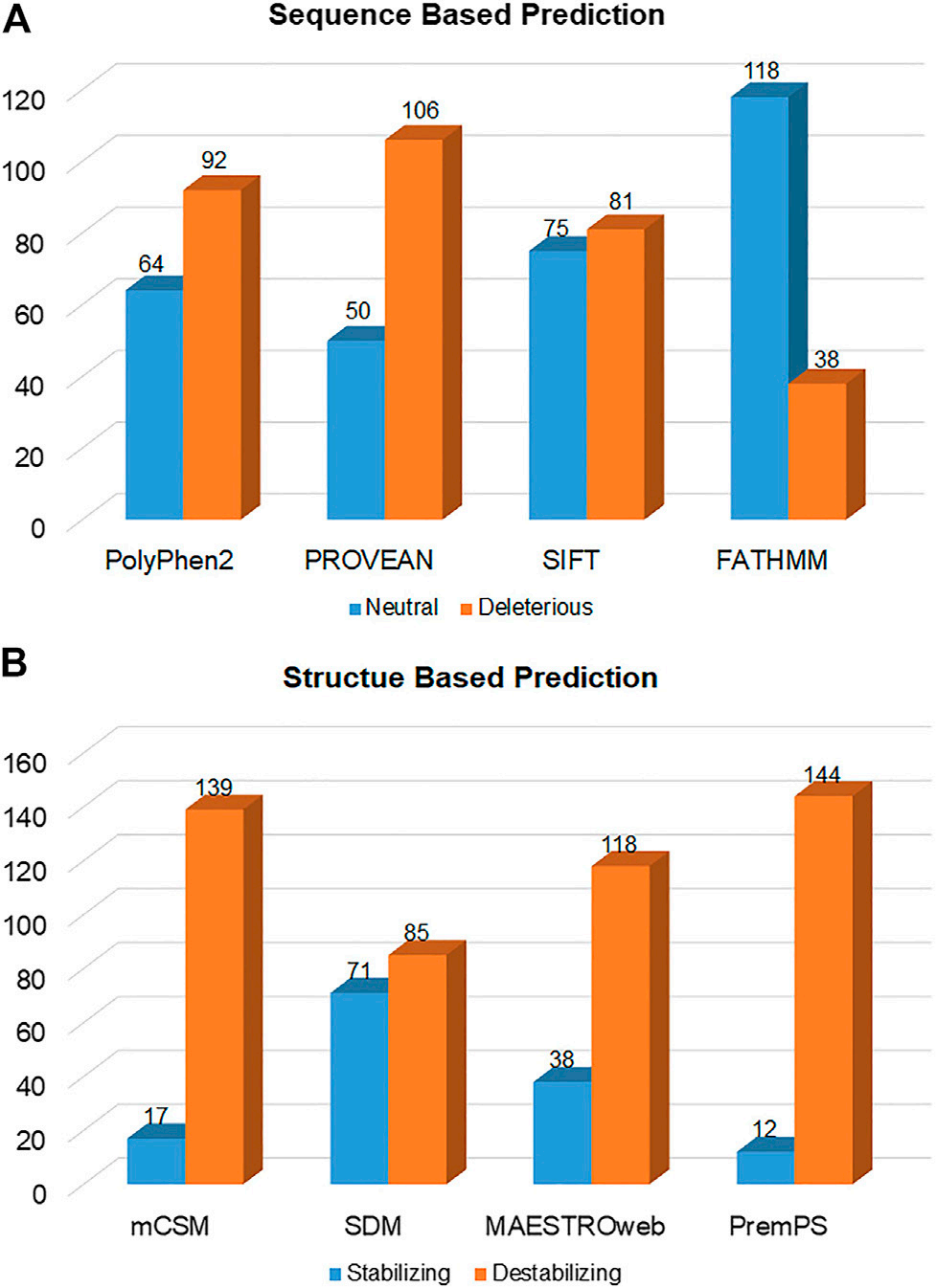
Deleterious and neutral mutations in SGK1 predicted by **(A)** sequence-based and **(B)** structure-based tools.

The substitutions which destabilize the structure of a protein are generally involved in various diseases (Ng and Henikoff, 2001; Petukh et al., 2015). The change in free energy during the unfolding of a kinetically stable protein is described by the ΔΔ*G* value. Sometimes a single amino acid substitution in proteins differentiates the free energy landscape between the mutant and WT protein. This variance in the free energy landscape is why a mutation affects the stability of a protein. Thermodynamically, the energy difference between a folded and unfolded protein can be considered as Δ*G* = Gu-Gf. The change of protein stability (ΔΔ*G*) and free energy landscape between mutant (Gm) and WT (Gw) is considered as ΔΔ*G* = Gm-Gw (Bowker-Kinley et al., 1998). A more positive ΔΔ*G* shows a destabilizing mutation, whereas a negative ΔΔ*G* indicates a more stabilizing mutation (Quan et al., 2016). We used various sequence-based predictors, i.e., PolyPhen2, PROVEAN, SIFT, and FATHMM, predicted that out of the 156 mutations, 92 (58.97%), 106 (67.94%), 81 (51.92%), and 38 (24.34%) were deleterious, respectively (Figure 3A), (Supplementary Table S1).

Structure-based predictors, i.e., mCSM, SDM, MAESTROweb, and PremPS, combine machine learning-based and biophysics-based approaches to determine the stability of mutants, calculating their free energy. This analysis showed that 139 (89.1%), 85 (54.48%), 118 (75.64%), and 144 (92.3%) mutations were destabilizing (Figure 3B; Supplementary Table S2). Mutations predicted to be deleterious by at least three different sequence-based and three different structure-based tools were selected to increase confidence levels. Here, 134 mutations were selected and then analyzed for their pathogenicity.

### Identification of Pathogenic Variations

**FIGURE 4:**
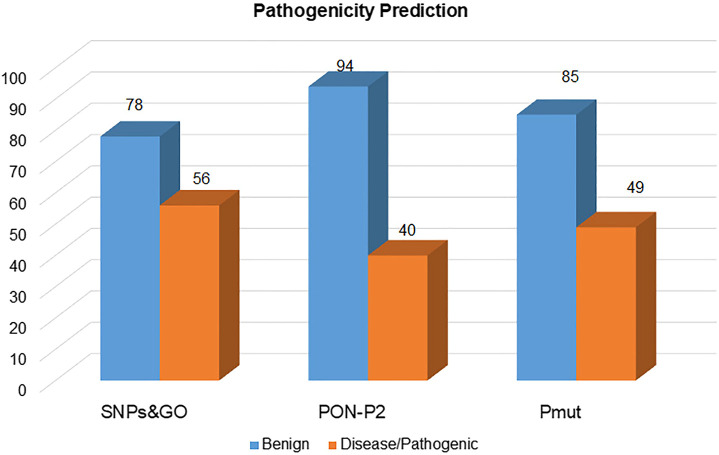
Pathogenic mutations in the SGK1 protein, identified using SNPs and GO, PON-P2, and PMut.

The selected mutations were predicted for their pathogenicity using the SNPs and GO, PON-P2, and PMut. From the 134 mutations, SNPs and GO, PON-P2, and PMut predicted 56, 40, and 49 mutations as pathogenic, respectively, (Figure 4). From these, only 20 mutations (P87R, K127M, R147W, L172W, G181V, Y186C, R198P, L230P, T256A, T256D, T256E, V278M, P296R, Y298A, I320N, D335V, R339W, G341A, P371R, and P374L) were predicted as pathogenic from all the prediction tools (Supplementary Table S3).

### Analysis of Evolutionarily Conserved Residues

**FIGURE 5:**
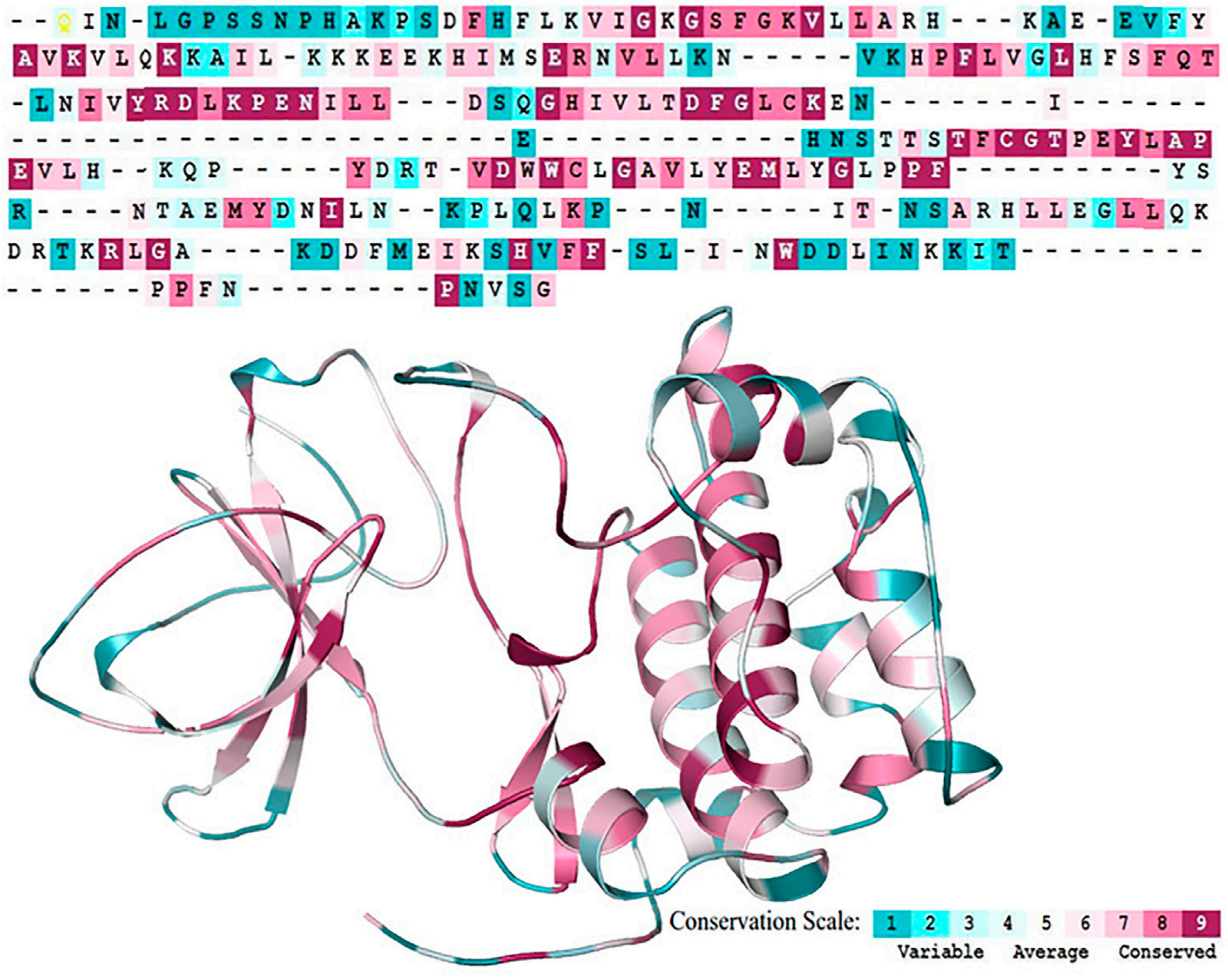
Sequence Conservation analysis of the SGK1 protein using ConSurf web server.

The overall integrity of a protein structure mainly depends on the conserved residues (Shakhnovich et al., 1996). Analysis of amino acid residue conservation in a protein structure is used to understand its importance and localized evolution. The propensity of an amino acid residue to mutate is subject to the degree of conservation (Ashkenazy et al., 2016). The SGK1 structure was analyzed to obtain the degree of conservation of each residue in the protein. The ConSurf analysis shows that the amino acids forming the central region of the SGK1 protein are highly conserved than those at the N- and C-termini (Figure 5). This signifies that any substitution in the central region of SGK1 will have more tendency to instability and thus its dysfunction in many diseases.

### Analysis of Aggregation Propensity

The solubility of a protein highly impacts its functionality (Balch et al., 2008; Ciryam et al., 2013). Diseases like Alzheimer’s (Thal et al., 2015), amyloidosis (Knowles et al., 2014), and Parkinson’s diseases (Knowles et al., 2014) are associated with protein aggregation. SODA predicts that out of the 20, 8 mutations decrease the solubility of the protein, whereas the other 12 increase the solubility of the SGK1 protein (Table 1). These mutants have a high tendency to get aggregate, thus their involvement in protein aggregation-associated disease progression. Finally, based on the functional importance and location of the mutations, three amino acid substitutions, i.e., K127M, T256A, and Y298A, were selected and studied in detail (Kobayashi and Cohen, 1999; Snyder et al., 2002; Boehmer et al., 2003).

**TABLE 1:** Aggregation propensity of SGK1 mutant proteins predicted through SODA.

Sequence	Helix	Strand	Aggregation	Disorder	SODA	Remark
Wild type	0.430	0.144	–4.596	0.084	N/A	N/A
P87R	–0.114	0.261	–1.542	0.311	–0.152	Less soluble
K127M	–0.218	0.174	–33.564	–0.131	–33.926	Less soluble
R147W	–3.366	2.907	–7.635	–0.009	–8.560	Less soluble
L172W	5.718	–4.082	–77.368	–0.05	–74.497	Less soluble
G181V	–2.478	1.961	–1.198	–0.002	–2.202	Less soluble
Y186C	2.108	–1.510	–32.643	0.009	–31.684	Less soluble
R198P	0.048	-0.834	7.485	–0.044	6.452	More soluble
L230P	–0.328	-0.405	4.512	0.013	2.851	More soluble
T256A	1.313	–0.680	0.973	–0.024	1.889	More soluble
T256D	–0.074	–0.231	3.709	–0.028	3.356	More soluble
T256E	0.374	-0.186	0.886	-0.013	1.548	More soluble
V278M	0.318	-0.243	1.443	–0.014	1.193	More soluble
P296R	0.024	–0.021	4.544	–0.136	4.649	More soluble
Y298A	–0.400	0.216	2.101	0.004	1.973	More soluble
I320N	–0.040	-0.258	1.305	0.022	0.752	More soluble
D335V	–0.252	0.123	–19.314	–0.283	–20.671	Less soluble
R339W	0.005	0.051	–3.193	–0.342	–4.114	Less soluble
G341A	1.228	–0.581	0.896	0.005	1.470	More soluble
P371R	0.912	–0.122	8.732	0.022	10.473	More soluble
P374L	0.894	–0.526	7.055	0.067	8.005	More soluble

### MD Simulations

#### Post-Dynamics Trajectory Analysis

**FIGURE 6:**
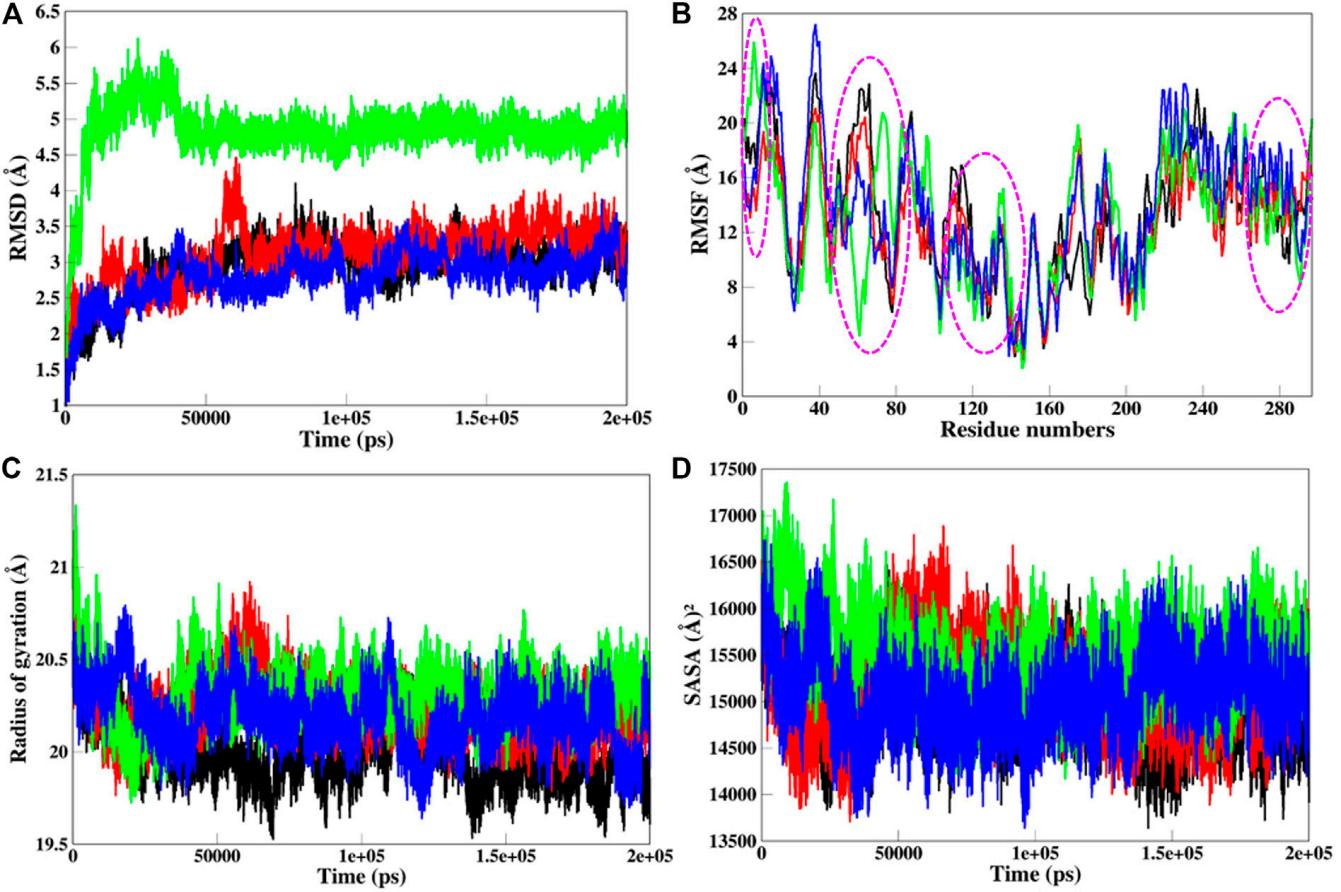
Structural dynamics of SGK1 WT (red), K127M (black), T256A (green), and Y298A (blue) mutants **(A)** RMSD, **(B)** RMSF, **(C)** Rg, and **(D)** SASA values across Cα backbone in Å of WT, K127M, T256A, and Y298A mutants calculated after 200 ns of MD trajectories.

MD simulations provide the platform for the comprehensive analysis of the effect of mutations on protein structure. Based on this, SGK1 mutations, i.e., K127M, T256A, and Y298A, were investigated using 200 ns simulated trajectories. Global protein stability and dynamics upon mutation were assessed through the time evolution of RMSD values. We computed the RMSDs for all four systems (SGK1 WT and its mutants) from the average simulated structure and plotted them for analysis (Figure 6A). All four systems achieved convergence after 60 ns of simulation. We observed a significant structural deviation in the T256A mutant compared with K127M, Y298A, and native SGK1. The RMSD values for T256A had a deviation of ∼0.2 nm from native SGK1 distributed throughout the simulation. The structures of Y298A and K127M had lower RMSD values compared with native SGK1. Although the mutants exhibited little deviation except T256A in the RMSD from the native structure. However, no substantial differences were observed in the structural snaps except the loop and N-terminal helices of superimposed SGK1-WT, K127M, T256A, and Y298A at every 50 ns during the simulation (Supplementary Figure S1). We plotted the dynamics of RMSD as the probability distribution function (PDF), which also illustrated a significant shift of ∼5 Å in T256A values with higher probability (Supplementary Figure S2A).

To explore the structural flexibility of active SGK1 and its mutants, we computed the RMSFs of each residue in the protein’s backbone (Figure 6B). SGK1 showed random fluctuations ranging from the N to the C termini, where the T256A mutant showed the highest fluctuations in most residues. Almost all residues in all systems exhibited a similar pattern of fluctuation; however, major changes were observed in the range of Q40-E120 amino acid residues. The mutant systems showed higher fluctuation compared to the native structure. In the T256A mutant, notable changes in fluctuation were observed for residues ∼50–80, whereas mutant K127M revealed several significant higher fluctuations for residues ∼10–30, ∼60–70, ∼110–120, and ∼230–240. The major peaks in the RMSF values of T256A were direct associated with the RMSD trend, where it majorly deviated from its initial position.

*R*g analysis exposes the structural compactness, stability, and folding mechanism of a protein structure (Lobanov et al., 2008). The folding mechanism and conformational behavior of the SGK1 structure and its mutants were studied by examining the time evolution of the *R*g values. We computed the *R*g values of native SGK1, K127M, T256A, and Y298A systems from the generated MD trajectories of 200 ns (Figure 6C). The *R*g of T256A and K127M exhibited the most deviation compared to WT and Y298A, especially after 30 ns. The Y298A structure also seems to be unfolded, showing several random fluctuations in its *Rg* values. The K127M mutant shows lower *Rg* thus higher compactness overall during the simulations. The PDF analysis also suggested a higher increase in the average *R*g values of T256A than WT SGK1, K127M, and Y298A suggested looseness of its conformational packing (Supplementary Figure S2B).

The SASA of a protein molecule is the surface area in contact with its surrounding solvent. The solvation power has a crucial role in maintaining the overall structure and folding of a protein. An inappropriate folded/unstable protein will not perform the function it supposes to be. So, it becomes crucial to study the folding behavior of the proteins upon mutations while exploring their SASA and packing density. The solvation power of a protein can be evaluated by explicit solvent models implemented in conventional MD simulation approaches. The time evolution of the SASA of native SGK1 and its mutant’s structure was computed and plotted (Figure 6D). This shows that T256A had higher SASA values than other systems, whereas K127M displayed a somewhat lower SASA than the native SGK1, agreeing with the *Rg* results. A clear shift in the distribution of the T256A SASA values in the PDF plot suggested a significant exposer of the buried residues of the protein thus its conformational shift (Supplementary Figure S2C).

#### Intramolecular Hydrogen Bond Analysis

**FIGURE 7:**
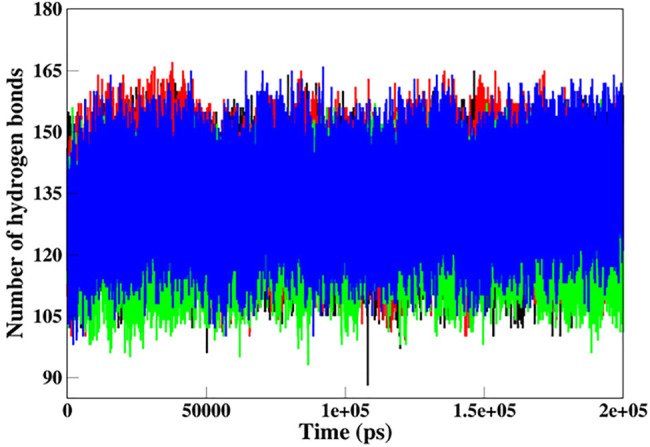
Intramolecular hydrogen bond analysis in SGK1 WT (red), K127M (black), T256A (green), and Y298A (blue).

Hydrogen bonds (H-bonds) are the most essential intramolecular interactions within a protein molecule (Myers and Pace, 1996). Since these interactions make major contributions to maintaining the stability of the protein structure, exploring the function of H-bonds offers crucial information about protein stability. Thus, we studied the time evolution of the number of intramolecular H-bonds in native and mutant SGK1 to understand structural stability during the simulation (Figure 7). The hydrogen bonding showed a little decrement in the number of intramolecular H-bonds in mutants throughout the simulation, especially in T256A.

#### Secondary Structure Analysis

**FIGURE 8:**
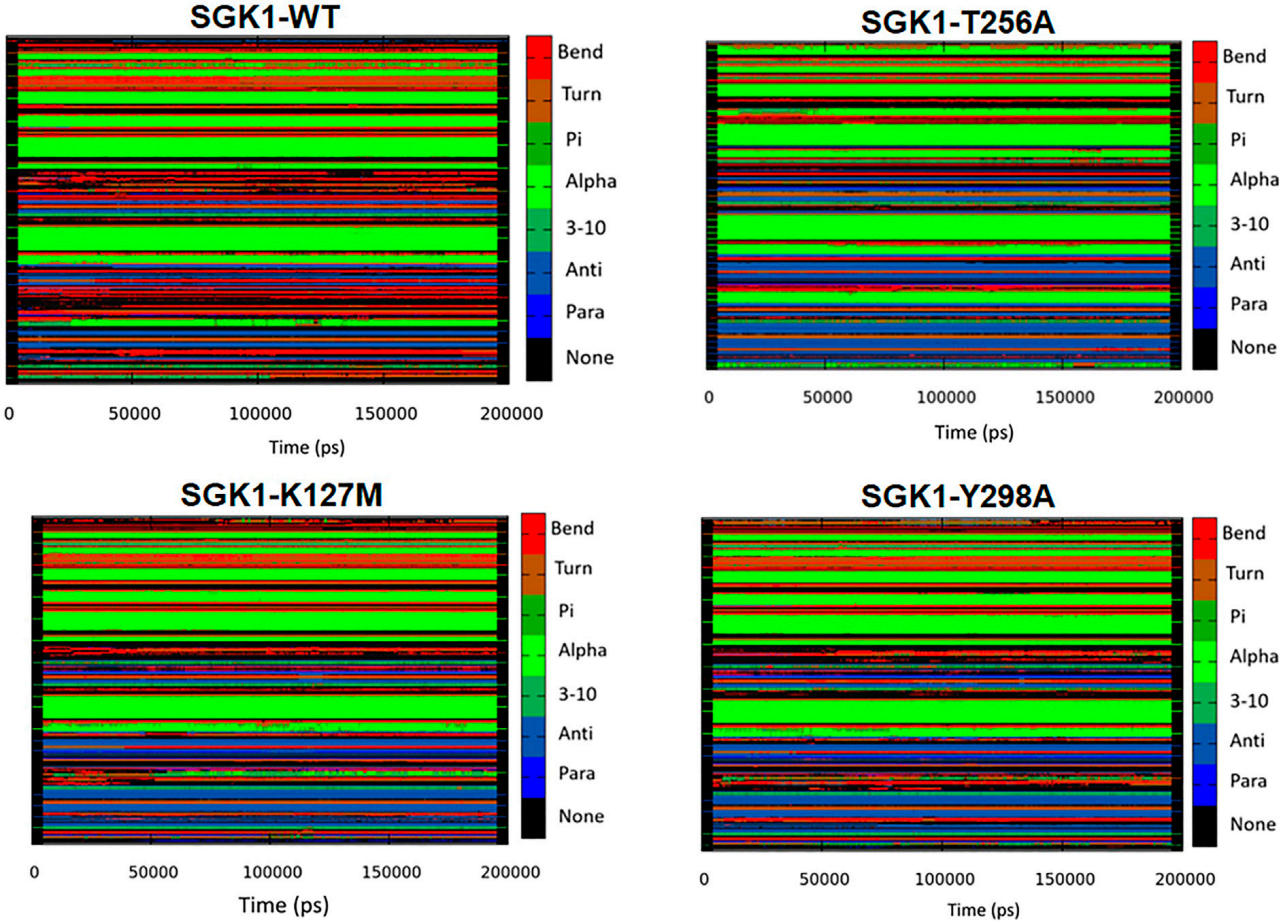
Secondary structural contents of SGK1 WT; T256A; K127M; and Y298A mutants.

The dynamics of secondary structure components in SGK1 and its mutants were evaluated from the MD trajectories of 200 ns. This further improves the understanding of the impact of mutations on the secondary structure of SGK1 during the simulations. The secondary structure components in SGK1, i.e., α-helix, β-sheets and turns, were split into specific residues for each time step. It was observed that the average number of residues that participated in the formation of secondary structure was somewhat decreased in T256A (Figure 8). This reduction was related to increases in the formation of turns and a slight decrease in α-helices and β-sheets (Table 2).

**TABLE 2:** Percentage of amino acid residues participating in the secondary structure of SGK1-WT, SGK1-T256A, SGK1-K127M, and SGK1-Y298A.

Protein system	α-helix	β-sheets	3_10_-helix	Turn	Bend	Other
SGK1-WT	25	24	4	10	13	21
SGK1-T256A	22	21	6	11	7	23
SGK1-K127M	28	25	7	16	11	24
SGK1-Y298A	27	26	4	12	9	20

### Distance Cross-Correlation Matrix

**FIGURE 9:**
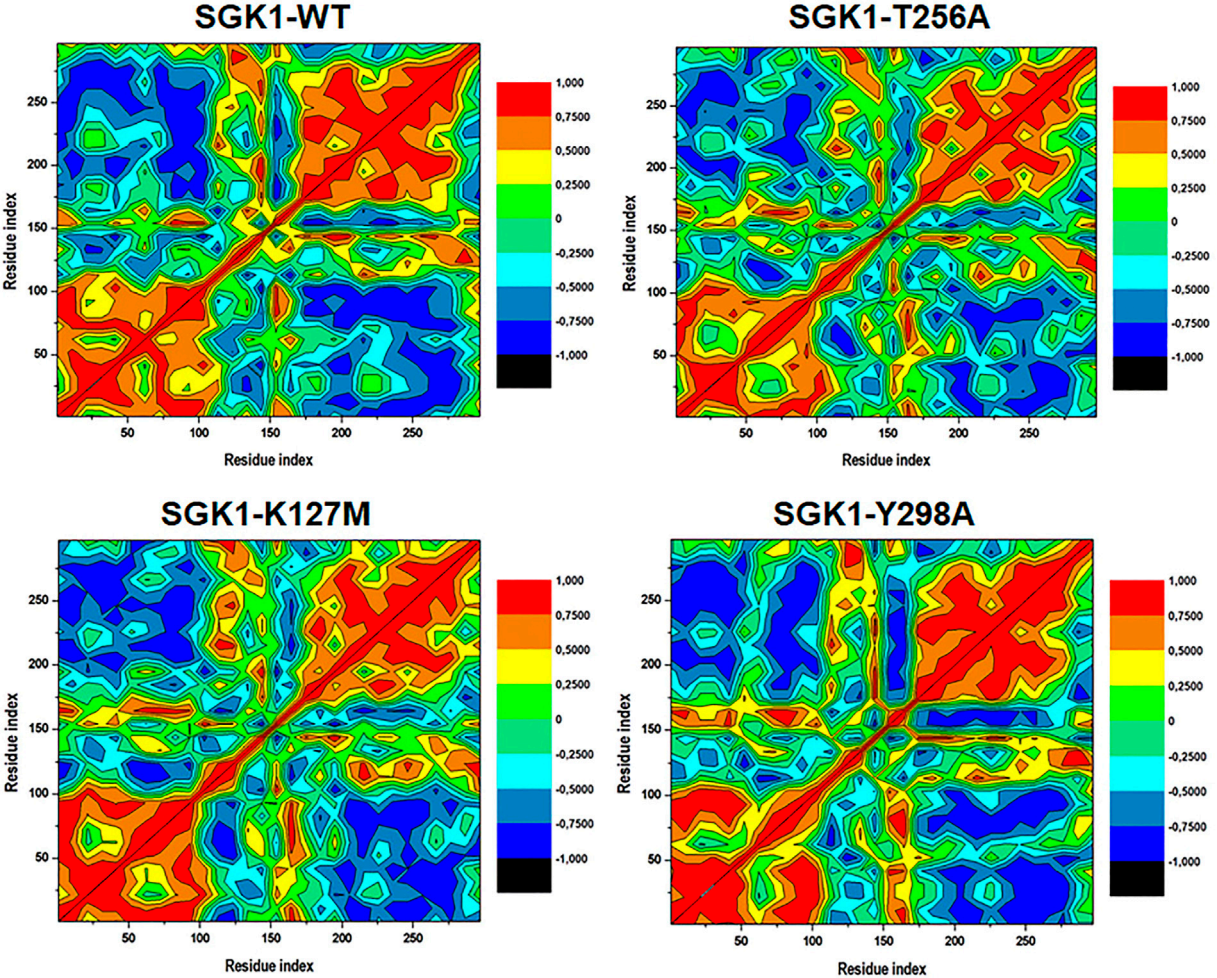
Dynamics cross-correlation matrices of SGK1-WT, K127M, T256A, and Y298A generated from 200 ns of MD trajectories.

Distance cross-correlation matrices were generated and evaluated for the native SGK1 and K127M, T256A, and Y298A mutants to determine correlated and anti-correlated movements in the protein’s structure (Figure 9). It was observed that SGK1 scattered into some populations through positive and negative correlations concerning residual movements. The movements in native SGK1 were quite equal in both positive and negative phases. In contrast, substantial variation was observed for mutants, especially in K127M and T256A, with more negative correlations. However, there was a slight positive correlation was observed in the K127M, majorly between 50 and 100 amino acid residues.

### PCA

**FIGURE 10:**
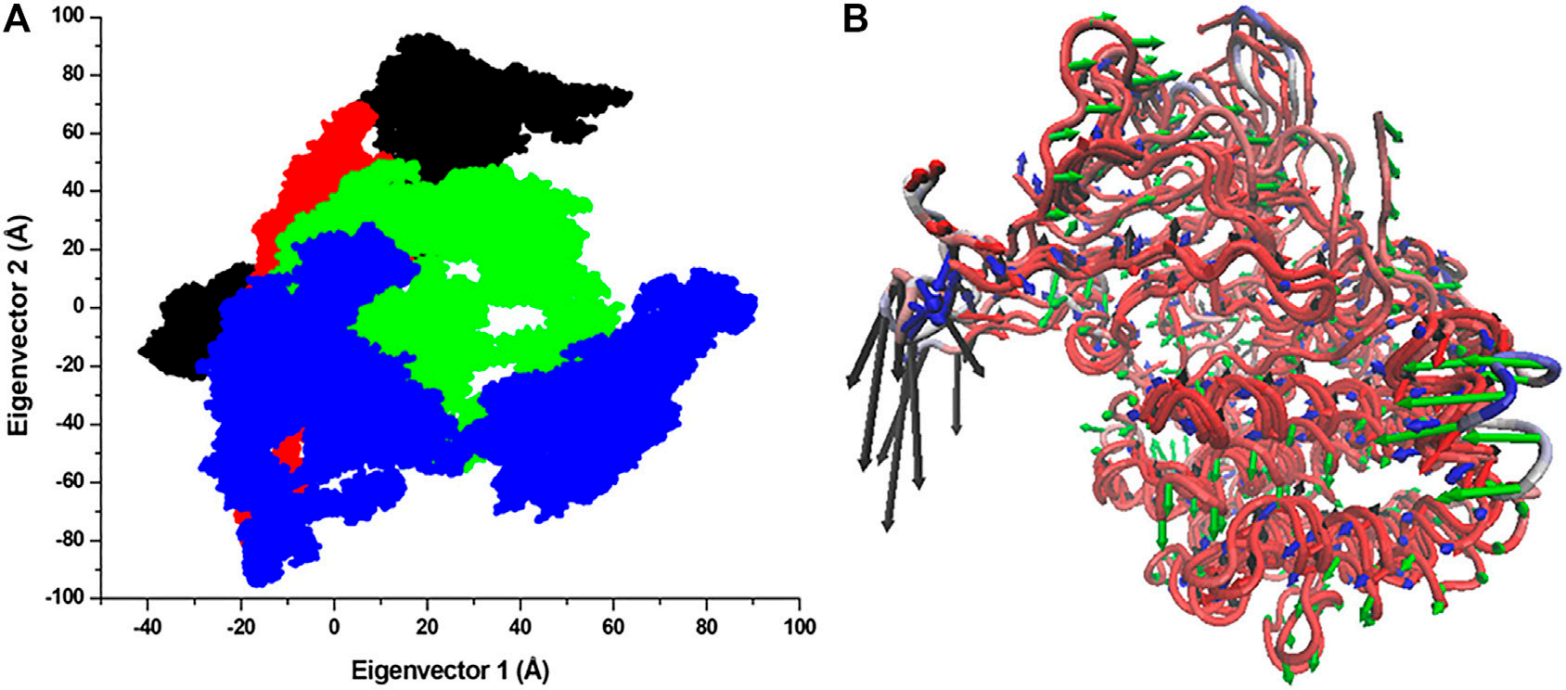
Conformational projection of SGK1 and its mutants. **(A)** Principal component analysis (PCA) of SGK1 WT (red), K127M (black), T256A (green), and Y298A (blue) mutants calculated after 200 ns of MD trajectories. **(B)** PC1 collective motions for the obtained predominant eigenvectors using PCA over the 200 ns MD trajectories for SGK1 WT, K127M, T256A, and Y298A mutants of SGK1.

The structural dynamics of a protein’s structure can be examined through its phase space performance. It has been exploited to observe the collective motions and conformational sampling of the proteins. PCA was performed using the essential dynamics approach to explore the conformational sampling and atomic motions of SGK1 and its mutants. PCA plots were constructed with PCs based on the first two eigenvectors (EVs) (Figure 10). The two-dimensional scatter plot reveals the conformational activities employed by SGK1 and its mutants (Figure 10A). At the same time, the PC1 motions in SGK1 and its mutants were assessed (Figure 10B). The 2D scatter plot (Figure 10A) indicates a prominent shift in the collective movements of mutant systems.

## Discussion

This study employs a systematic computational approach based on various biophysical algorithms to study the impact of mutations on SGK1 structure and function for understanding their association with multiple diseases, such as cancer and neurodegeneration. Sequence and structure-based analyses suggested that 134 mutations were deleterious out of a total of 156 mutations present in SGK1. Here, 20 mutations were found to be pathogenic, predicted through the pathogenicity study. Further, aggregation tendency analysis showed that only 8 mutations in SGK1 were less soluble and tended to form aggregates. The ConSurf analysis showed that the amino acids forming the middle segment of the SGK1 protein are highly conserved than those at the N- and C-termini. Finally, based on the functional importance (Kobayashi and Cohen, 1999; Snyder et al., 2002; Boehmer et al., 2003) and location of the mutations, three amino acid substitutions, i.e., K127M, T256A, and Y298A, were selected and studied in detail. A detailed analysis of these mutations was performed, with the help of MD simulation studies for 200 ns, followed by DCCM and PCA studies.

In MD simulations, the RMSD of T256A reflects a stability change in the structure and indicates the deleterious impact of the mutation on SGK1. A major deviation was also observed in the K127M mutant intramolecularly at the 50 ns time step, suggesting a significant impact of the mutation on the ATP binding site. The RMSF analysis suggested that the residual fluctuations in all the mutants deviated from the native structure. These deviations in RMSFs reflect the impact of deleterious mutations on the SGK1 structure. While evaluating the compactness of SGK1 and its mutants, the *R*g showed reduced stability of all three mutants during the course of simulations, suggesting structural lethality in SGK1 resulting from the induced mutations. The notable differences in SASA values of the mutants revealed that relocation of amino acid residues from accessible areas to buried regions, or vice versa, may take place and can cause significant changes to protein stability. Together, these explanations reveal that alterations in the SGK1 structure are associated with the induced mutations.

The intramolecular hydrogen bond analysis showed that the number of H-bonds in the mutants fluctuated compared with the stable number of H-bonds in the native SGK1. This fluctuation in H-bonds in the mutants indicates the impact of induced mutations and their capability to obliterate H-bond formation in SGK1. Secondary structure analysis showed that α-helices and β-sheets were increased in SGK1 after K127M and Y298A mutations, while a slight decrease was detected in the percentage of bends. This residual reduction in α-helices and β-strands of T256A suggests a loss in structure, thus its dysfunction. In DCCM, the correlated and anti-correlated movements in native SGK1 and Y298A appear to be more similar compared to K127M and T256A, suggesting SGK1 altered activity in K127M and T256A mutants. PCA indicated that K127M has highly positive correlated fluctuations on both EVs, signifying its altered movements. Whereas with T256A and Y298A mutants, noticeable positively correlated progress was only observed on EV1. Overall, the PCA suggests that K127, T256A, and Y298A mutations cause large instabilities in the SGK1 structural movements during the simulation.

## Conclusion

Single amino acid substitutions are among the most frequent genetic variations associated with numerous diseases, including cancer and neurodegeneration. Extensive analysis of amino acid substitutions helps to understand disease mechanisms and find effective treatments. Here, we have extensively analyzed the effects of known mutations in SGK1 protein on its structure and function. Sequence and structure-based analyses suggest that out of 156 mutations present SGK1, 134 mutations were deleterious and destabilizing. Here, 20 mutations were found to be pathogenic, predicted through the pathogenicity study. Further, aggregation tendency analysis showed that only 8 mutations in SGK1 were less soluble and tended to form aggregates, resulting in protein dysfunction, thus might involve in aggregation-associated disease progression. Finally, based on the functional importance and location of the mutations, three amino acid substitutions, i.e., K127M, T256A, and Y298A, were selected and studied in detail. A detailed analysis of these mutations was performed, with the help of MD simulation studies for 200 ns, followed by PCA and DCCM studies. MD simulations result suggested that the pathogenic impact of these mutations may arise due to structural modifications in SGK1. MD simulation analyses, including RMSD, RMSF, Rg, SASA, DCCM, and PCA, indicated that SGK1 undergoes substantial conformational changes due to mutations, especially in the case of K127 and T256A. This study provides a comprehensive understanding of the mutations in SGK1 and their possible consequences for disease progression.

## Data Availability

The original contributions presented in the study are included in the article/Supplementary Material, further inquiries can be directed to the corresponding authors.

## References

[B1] Adzhubei I. A. Schmidt S. Peshkin L. Ramensky V. E. Gerasimova A. Bork P. (2010). A Method and Server for Predicting Damaging Missense Mutations. Nat. Methods 7, 248–249. 10.1038/nmeth0410-248 20354512 PMC2855889

[B2] Amir M. Kumar V. Mohammad T. Dohare R. Hussain A. Rehman M. T. (2019a). Investigation of Deleterious Effects of nsSNPs in the POT1 Gene: a Structural Genomics‐based Approach to Understand the Mechanism of Cancer Development. J. Cel Biochem 120, 10281–10294. 10.1002/jcb.28312 30556179

[B3] Amir M. Mohammad T. Kumar V. AlAjmi M. F. Rehman M. T. Hussain A. (2019b). Structural Analysis and Conformational Dynamics of STN1 Gene Mutations Involved in Coat Plus Syndrome. Front. Mol. Biosci. 6, 41. 10.3389/fmolb.2019.00041 31245382 PMC6581698

[B4] Andersen H. C. (1983). Rattle: A “Velocity” Version of the Shake Algorithm for Molecular Dynamics Calculations. J. Comput. Phys. 52, 24–34. 10.1016/0021-9991(83)90014-1

[B5] Ashkenazy H. Abadi S. Martz E. Chay O. Mayrose I. Pupko T. (2016). ConSurf 2016: an Improved Methodology to Estimate and Visualize Evolutionary Conservation in Macromolecules. Nucleic Acids Res. 44, W344–W350. 10.1093/nar/gkw408 27166375 PMC4987940

[B6] Baak J. P. A. Path F. R. C. Hermsen M. A. J. A. Meijer G. Schmidt J. Janssen E. A. M. (2003). Genomics and Proteomics in Cancer. Eur. J. Cancer 39, 1199–1215. 10.1016/s0959-8049(03)00265-x 12763207

[B7] Balch W. E. Morimoto R. I. Dillin A. Kelly J. W. (2008). Adapting Proteostasis for Disease Intervention. Science 319, 916–919. 10.1126/science.1141448 18276881

[B8] Berman H. M. Westbrook J. Feng Z. Gilliland G. Bhat T. N. Weissig H. (2000). The Protein Data Bank. Nucleic Acids Res. 28, 235–242. 10.1093/nar/28.1.235 10592235 PMC102472

[B9] Boehmer C. Henke G. Schniepp R. Palmada M. Rothstein J. D. Bröer S. (2003). Regulation of the Glutamate Transporter EAAT1 by the Ubiquitin Ligase Nedd4-2 and the Serum and Glucocorticoid-Inducible Kinase Isoforms SGK1/3 and Protein Kinase B. J. Neurochem. 86, 1181–1188. 10.1046/j.1471-4159.2003.01937.x 12911626

[B10] Bowker-Kinley M. M. Davis W. I. Wu P. Harris R. A. Popov K. M. (1998). Evidence for Existence of Tissue-specific Regulation of the Mammalian Pyruvate Dehydrogenase Complex. Biochem. J. 329 (Pt 1), 191–196. 10.1042/bj3290191 9405293 PMC1219031

[B11] Capriotti E. Calabrese R. Fariselli P. Martelli P. L. Altman R. B. Casadio R. (2013). WS-SNPs&GO: a Web Server for Predicting the Deleterious Effect of Human Protein Variants Using Functional Annotation. BMC genomics 14 (Suppl. 3), S6. 10.1186/1471-2164-14-S3-S6 PMC3665478 23819482

[B12] Chen Y. Lu H. Zhang N. Zhu Z. Wang S. Li M. (2020). PremPS: Predicting the Impact of Missense Mutations on Protein Stability. Plos Comput. Biol. 16, e1008543. 10.1371/journal.pcbi.1008543 33378330 PMC7802934

[B13] Choi Y. Chan A. P. (2015). PROVEAN Web Server: a Tool to Predict the Functional Effect of Amino Acid Substitutions and Indels. Bioinformatics 31, 2745–2747. 10.1093/bioinformatics/btv195 25851949 PMC4528627

[B14] Choudhury A. Mohammad T. Samarth N. Hussain A. Rehman M. T. Islam A. (2021). Structural Genomics Approach to Investigate Deleterious Impact of nsSNPs in Conserved Telomere Maintenance Component 1. Sci. Rep. 11, 10202–10213. 10.1038/s41598-021-89450-7 33986331 PMC8119478

[B15] Chun S. Fay J. C. (2009). Identification of Deleterious Mutations within Three Human Genomes. Genome Res. 19, 1553–1561. 10.1101/gr.092619.109 19602639 PMC2752137

[B16] Ciryam P. Tartaglia G. G. Morimoto R. I. Dobson C. M. Vendruscolo M. (2013). Widespread Aggregation and Neurodegenerative Diseases Are Associated with Supersaturated Proteins. Cel Rep. 5, 781–790. 10.1016/j.celrep.2013.09.043 PMC3883113 24183671

[B17] David C. C. Jacobs D. J. (2014). “Principal Component Analysis: a Method for Determining the Essential Dynamics of Proteins,” in Protein Dynamics (Totowa, NJ: Springer), 193–226. 10.1007/978-1-62703-658-0_11 PMC4676806 24061923

[B18] DeLano W. L. (2002). Pymol: An Open-Source Molecular Graphics Tool. CCP4 Newsl. Protein Crystallogr. 40, 82–92.

[B19] Eapen M. S. Sharma P. Thompson I. E. Lu W. Myers S. Hansbro P. M. (2019). Heparin-binding Epidermal Growth Factor (HB-EGF) Drives EMT in Patients with COPD: Implications for Disease Pathogenesis and Novel Therapies. Lab. Invest. 99, 150–157. 10.1038/s41374-018-0146-0 30451982

[B20] Fatima S. Mohammad T. Jairajpuri D. S. Rehman M. T. Hussain A. Samim M. (2019). Identification and Evaluation of Glutathione Conjugate Gamma-L-Glutamyl-L-Cysteine for Improved Drug-Delivery to the Brain. J. Biomol. Struct. Dyn. 38 (12), 3610–3620. 10.1080/07391102.2019.1664937 31496427

[B21] Habib I. Khan S. Mohammad T. Hussain A. Alajmi M. F. Rehman T. (2021). Impact of Non-synonymous Mutations on the Structure and Function of Telomeric Repeat Binding Factor 1. J. Biomol. Struct. Dyn., 1–14. 10.1080/07391102.2021.1922313 33982644

[B22] Henke G. Maier G. Wallisch S. Boehmer C. Lang F. (2004). Regulation of the Voltage Gated K+ Channel Kv1.3 by the Ubiquitin Ligase Nedd4-2 and the Serum and Glucocorticoid Inducible Kinase SGK1. J. Cel. Physiol. 199, 194–199. 10.1002/jcp.10430 15040001

[B23] Hubbard T. Barker D. Birney E. Cameron G. Chen Y. Clark L. (2002). The Ensembl Genome Database Project. Nucleic Acids Res. 30, 38–41. 10.1093/nar/30.1.38 11752248 PMC99161

[B24] Humphrey W. Dalke A. Schulten K. (1996). VMD: Visual Molecular Dynamics. J. Mol. graphics 14, 33–38. 10.1016/0263-7855(96)00018-5 8744570

[B25] Knowles T. P. J. Vendruscolo M. Dobson C. M. (2014). The Amyloid State and its Association with Protein Misfolding Diseases. Nat. Rev. Mol. Cel Biol 15, 384–396. 10.1038/nrm3810 24854788

[B26] Kobayashi T. Cohen P. (1999). Activation of Serum- and Glucocorticoid-Regulated Protein Kinase by Agonists that Activate Phosphatidylinositide 3-kinase Is Mediated by 3-phosphoinositide-dependent Protein Kinase-1 (PDK1) and PDK2. Biochem. J. 339, 319–328. 10.1042/bj3390319 10191262 PMC1220160

[B27] Kumar P. Henikoff S. Ng P. C. (2009). Predicting the Effects of Coding Non-synonymous Variants on Protein Function Using the SIFT Algorithm. Nat. Protoc. 4, 1073–1081. 10.1038/nprot.2009.86 19561590

[B28] Laimer J. Hofer H. Fritz M. Wegenkittl S. Lackner P. (2015). MAESTRO - Multi Agent Stability Prediction upon point Mutations. BMC bioinformatics 16, 116. 10.1186/s12859-015-0548-6 25885774 PMC4403899

[B29] Lang F. Perrotti N. Stournaras C. (2010). Colorectal Carcinoma Cells-Regulation of Survival and Growth by SGK1. Int. J. Biochem. Cel Biol. 42, 1571–1575. 10.1016/j.biocel.2010.05.016 20541034

[B30] Lobanov M. Y. Bogatyreva N. S. Galzitskaya O. V. (2008). Radius of Gyration as an Indicator of Protein Structure Compactness. Mol. Biol. 42, 623–628. 10.1134/s0026893308040195 18856071

[B31] López-Ferrando V. Gazzo A. de la Cruz X. Orozco M. Gelpí J. L. (2017). PMut: a Web-Based Tool for the Annotation of Pathological Variants on Proteins, 2017 Update. Nucleic Acids Res. 45, W222–w228. 10.1093/nar/gkx313 28453649 PMC5793831

[B32] Lu Y. Chan Y. T. Tan H. Y. Li S. Wang N. Feng Y. (2020). Epigenetic Regulation in Human Cancer: the Potential Role of Epi-Drug in Cancer Therapy. Mol. Cancer 19, 79–16. 10.1186/s12943-020-01197-3 32340605 PMC7184703

[B33] Mahmood M. Q. Ward C. Muller H. K. Sohal S. S. Walters E. H. (2017). Epithelial Mesenchymal Transition (EMT) and Non-small Cell Lung Cancer (NSCLC): a Mutual Association with Airway Disease. Med. Oncol. 34, 45–10. 10.1007/s12032-017-0900-y 28197929

[B34] Mohammad T. Khan F. I. Lobb K. A. Islam A. Ahmad F. Hassan M. I. (2019). Identification and Evaluation of Bioactive Natural Products as Potential Inhibitors of Human Microtubule Affinity-Regulating Kinase 4 (MARK4). J. Biomol. Struct. Dyn. 37, 1813–1829. 10.1080/07391102.2018.1468282 29683402

[B35] Myers J. K. Pace C. N. (1996). Hydrogen Bonding Stabilizes Globular Proteins. Biophysical J. 71, 2033–2039. 10.1016/s0006-3495(96)79401-8 PMC1233669 8889177

[B36] Naqvi A. A. T. Mohammad T. Hasan G. M. Hassan M. I. (2018). Advancements in Docking and Molecular Dynamics Simulations towards Ligand-Receptor Interactions and Structure-Function Relationships. Ctmc 18, 1755–1768. 10.2174/1568026618666181025114157 30360721

[B37] Ng P. C. Henikoff S. (2001). Predicting Deleterious Amino Acid Substitutions. Genome Res. 11, 863–874. 10.1101/gr.176601 11337480 PMC311071

[B38] Ng P. C. Henikoff S. (2006). Predicting the Effects of Amino Acid Substitutions on Protein Function. Annu. Rev. Genom. Hum. Genet. 7, 61–80. 10.1146/annurev.genom.7.080505.115630 16824020

[B39] Ng P. C. Henikoff S. (2003). SIFT: Predicting Amino Acid Changes that Affect Protein Function. Nucleic Acids Res. 31, 3812–3814. 10.1093/nar/gkg509 12824425 PMC168916

[B40] Niroula A. Urolagin S. Vihinen M. (2015). PON-P2: Prediction Method for Fast and Reliable Identification of Harmful Variants. PloS one 10, e0117380. 10.1371/journal.pone.0117380 25647319 PMC4315405

[B41] O'Keeffe B. A. Cilia S. Maiyar A. C. Vaysberg M. Firestone G. L. (2013). The Serum- and Glucocorticoid-Induced Protein Kinase-1 (Sgk-1) Mitochondria Connection: Identification of the IF-1 Inhibitor of the F1F0-ATPase as a Mitochondria-specific Binding Target and the Stress-Induced Mitochondrial Localization of Endogenous Sgk-1. Biochimie 95, 1258–1265. 10.1016/j.biochi.2013.01.019 23402912 PMC3684451

[B42] Overington J. Donnelly D. Johnson M. S. Šali A. Blundell T. L. (1992). Environment-specific Amino Acid Substitution Tables: Tertiary Templates and Prediction of Protein Folds. Protein Sci. 1, 216–226. 10.1002/pro.5560010203 1304904 PMC2142193

[B43] Paladin L. Piovesan D. Tosatto S. C. E. (2017). SODA: Prediction of Protein Solubility from Disorder and Aggregation Propensity. 45, W236–W240. 10.1093/nar/gkx412 PMC7059794 28505312

[B44] Pandurangan A. P. Ochoa-Montaño B. Ascher D. B. Blundell T. L. (2017). SDM: a Server for Predicting Effects of Mutations on Protein Stability. Nucleic Acids Res. 45, W229–w235. 10.1093/nar/gkx439 28525590 PMC5793720

[B45] Papaleo E. Mereghetti P. Fantucci P. Grandori R. De Gioia L. (2009). Free-energy Landscape, Principal Component Analysis, and Structural Clustering to Identify Representative Conformations from Molecular Dynamics Simulations: the Myoglobin Case. J. Mol. graphics Model. 27, 889–899. 10.1016/j.jmgm.2009.01.006 19264523

[B46] Petukh M. Kucukkal T. G. Alexov E. (2015). On Human Disease-Causing Amino Acid Variants: Statistical Study of Sequence and Structural Patterns. Hum. Mutat. 36, 524–534. 10.1002/humu.22770 25689729 PMC4409542

[B47] Pires D. E. V. Ascher D. B. Blundell T. L. (2014). mCSM: Predicting the Effects of Mutations in Proteins Using Graph-Based Signatures. Bioinformatics (Oxford, England) 30, 335–342. 10.1093/bioinformatics/btt691 PMC3904523 24281696

[B48] Quan L. Lv Q. Zhang Y. (2016). STRUM: Structure-Based Prediction of Protein Stability Changes upon Single-point Mutation. Bioinformatics 32, 2936–2946. 10.1093/bioinformatics/btw361 27318206 PMC5039926

[B49] Ramensky V. Bork P. Sunyaev S. (2002). Human Non-synonymous SNPs: Server and Survey. Nucleic Acids Res. 30, 3894–3900. 10.1093/nar/gkf493 12202775 PMC137415

[B50] Roe D. R. Cheatham T. E. III (2013). PTRAJ and CPPTRAJ: Software for Processing and Analysis of Molecular Dynamics Trajectory Data. J. Chem. Theor. Comput. 9, 3084–3095. 10.1021/ct400341p 26583988

[B51] Sang Y. Kong P. Zhang S. Zhang L. Cao Y. Duan X. (2021). SGK1 in Human Cancer: Emerging Roles and Mechanisms. Front. Oncol. 10, 2987. 10.3389/fonc.2020.608722 PMC7851074 33542904

[B52] Sekido Y. (2010). Genomic Abnormalities and Signal Transduction Dysregulation in Malignant Mesothelioma Cells. Cancer Sci. 101, 1–6. 10.1111/j.1349-7006.2009.01336.x 19793348 PMC11159396

[B53] Shakhnovich E. Abkevich V. Ptitsyn O. (1996). Conserved Residues and the Mechanism of Protein Folding. Nature 379, 96–98. 10.1038/379096a0 8538750

[B54] Sherry S. T. Ward M. H. Kholodov M. Baker J. Phan L. Smigielski E. M. (2001). dbSNP: the NCBI Database of Genetic Variation. Nucleic Acids Res. 29, 308–311. 10.1093/nar/29.1.308 11125122 PMC29783

[B55] Shihab H. A. Gough J. Cooper D. N. Stenson P. D. Barker G. L. A. Edwards K. J. (2013). Predicting the Functional, Molecular, and Phenotypic Consequences of Amino Acid Substitutions Using Hidden Markov Models. Hum. Mutat. 34, 57–65. 10.1002/humu.22225 23033316 PMC3558800

[B56] Snyder P. M. Olson D. R. Thomas B. C. (2002). Serum and Glucocorticoid-Regulated Kinase Modulates Nedd4-2-Mediated Inhibition of the Epithelial Na+Channel. J. Biol. Chem. 277, 5–8. 10.1074/jbc.c100623200 11696533

[B57] Thal D. R. Walter J. Saido T. C. Fändrich M. (2015). Neuropathology and Biochemistry of Aβ and its Aggregates in Alzheimer's Disease. Acta Neuropathol. 129, 167–182. 10.1007/s00401-014-1375-y 25534025

[B58] Umair M. Khan S. Mohammad T. Shafie A. Anjum F. Islam A. (2021). Impact of Single Amino Acid Substitution on the Structure and Function of TANK‐binding Kinase‐1. J. Cell. Biochem 122(10), 1475–1490. 10.1002/jcb.30070 34237165

[B59] Waldegger S. Erdel M. Nagl U. O. Barth P. Raber G. Steuer S. (1998). Genomic Organization and Chromosomal Localization of the HumanSGKProtein Kinase Gene. Genomics 51, 299–302. 10.1006/geno.1998.5258 9722955

[B60] Zhao B. Lehr R. Smallwood A. M. Ho T. F. Maley K. Randall T. (2007). Crystal Structure of the Kinase Domain of Serum and Glucocorticoid-Regulated Kinase 1 in Complex with AMP-PNP. Protein Sci. 16, 2761–2769. 10.1110/ps.073161707 17965184 PMC2222817

