# Supplementary material for: Impact of Deleterious Mutations on Structure, Function and Stability of Serum/Glucocorticoid Regulated Kinase 1: A Gene to Diseases Correlation
Source: Front Mol Biosci. 2021 Nov 3;8:780284. doi: 10.3389/fmolb.2021.780284 (PMC8597711; doi:10.3389/fmolb.2021.780284)
Supplement: Supplementary file 1 [file DataSheet1.docx]

*Supplementary*

**Impact of Deleterious Mutations on Structure, Function and Stability of Serum/Glucocorticoid Regulated Kinase 1: A Gene to Diseases Correlation**

Mohamed F. AlAjmi^1^, Shama Khan^2^, Arunabh Choudhury^3^, Taj Mohammad^4^, Saba Noor^4^, Afzal Hussain^1^, Wenying Lu^5^, Mathew Suji Eapen^5^, Vrushali Chimankar^6,7^, Philip M Hansbro^7^, Sukhwinder Singh Sohal^5^, Abdelbaset Mohamed Elasbali^8,*^ and Md. Imtaiyaz Hassan^4,*^

^1^Department of Pharmacognosy, College of Pharmacy, King Saud University, Riyadh, Saudi Arabia.

^2^Drug Discovery and Development Centre (H3D), University of Cape Town, Rondebosch 7701, South Africa.

^3^Department of Computer Science, Jamia Millia Islamia, New Delhi – 110025, India.

**^4^**Centre for Interdisciplinary Research in Basic Sciences, Jamia Millia Islamia, Jamia Nagar, New Delhi 110025, INDIA.

^5^Respiratory Translational Research Group, Department of Laboratory Medicine, School of Health Sciences, College of Health and Medicine, University of Tasmania, Newnham, Launceston, Tasmania 7248, Australia.

^6^Centre for Inflammation, Centenary Institute and University of Technology Sydney, School of Life Sciences, Faculty of Science, Sydney, NSW, Australia.

^7^Priority Research Centre for Healthy Lungs & Hunter Medical Research Institute, The University of Newcastle, Newcastle, NSW 2305, Australia.

^8^Clinical Laboratory Science, College of Applied Sciences-Qurayyat, Jouf University, Saudi Arabia.

*Correspondence: [aeelasbali@ju.edu.sa](mailto:aeelasbali@ju.edu.sa) (A.M.E.); [mihassan@jmi.ac.in](mailto:mihassan@jmi.ac.in) (M. I. H.)

**
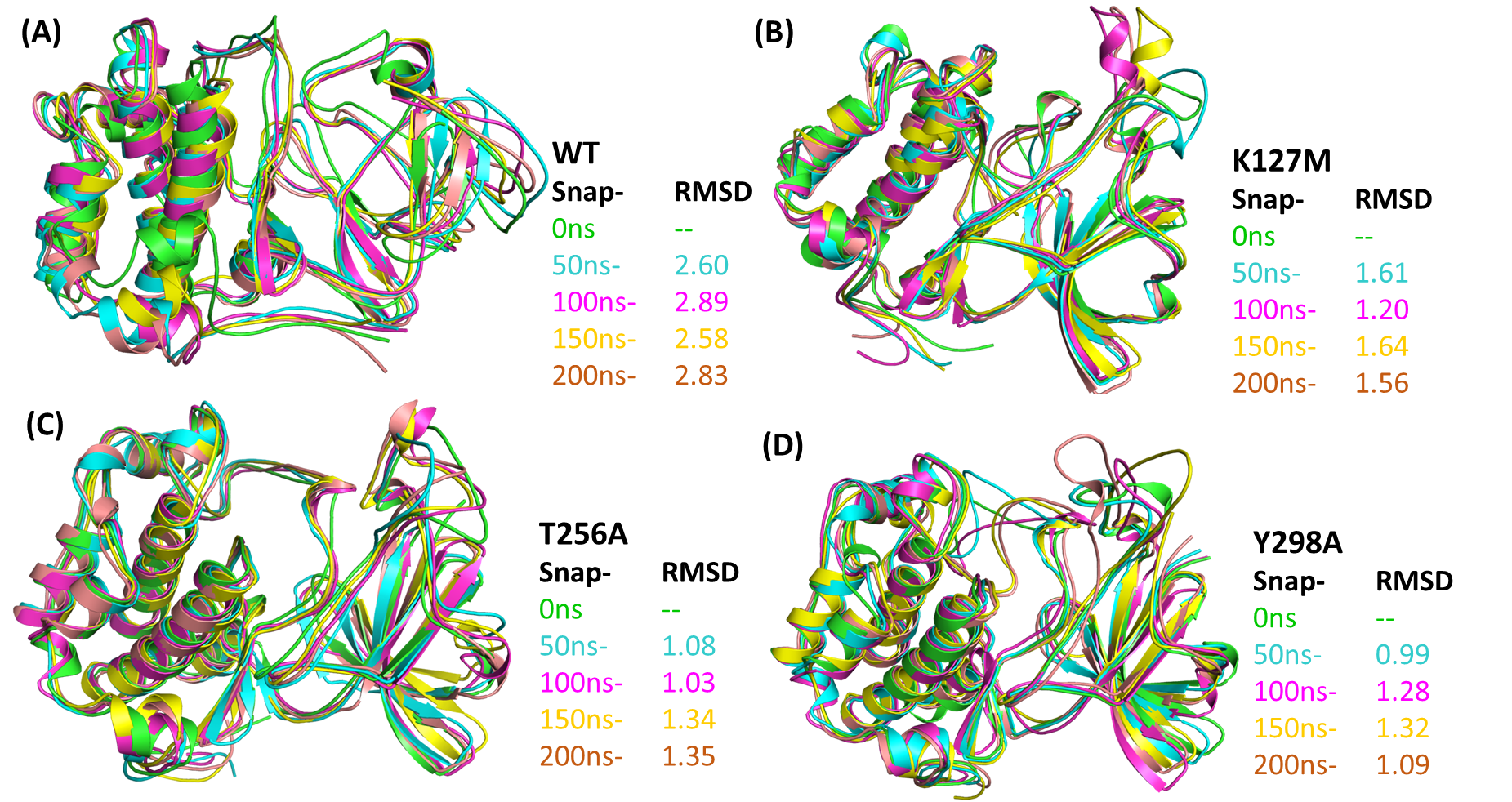
**

**Figure S1:** Structural snapshots of SGK1 (A) WT, (B) K127M, (C) T256A, and (D) Y298A at an interval of 50 ns from 0 to 200 ns of simulation. Corresponding panels show the RMSD of structural alignment of SGK1 at different time. Structures were drawn using PyMOL (<https://pymol.org/2>).

**
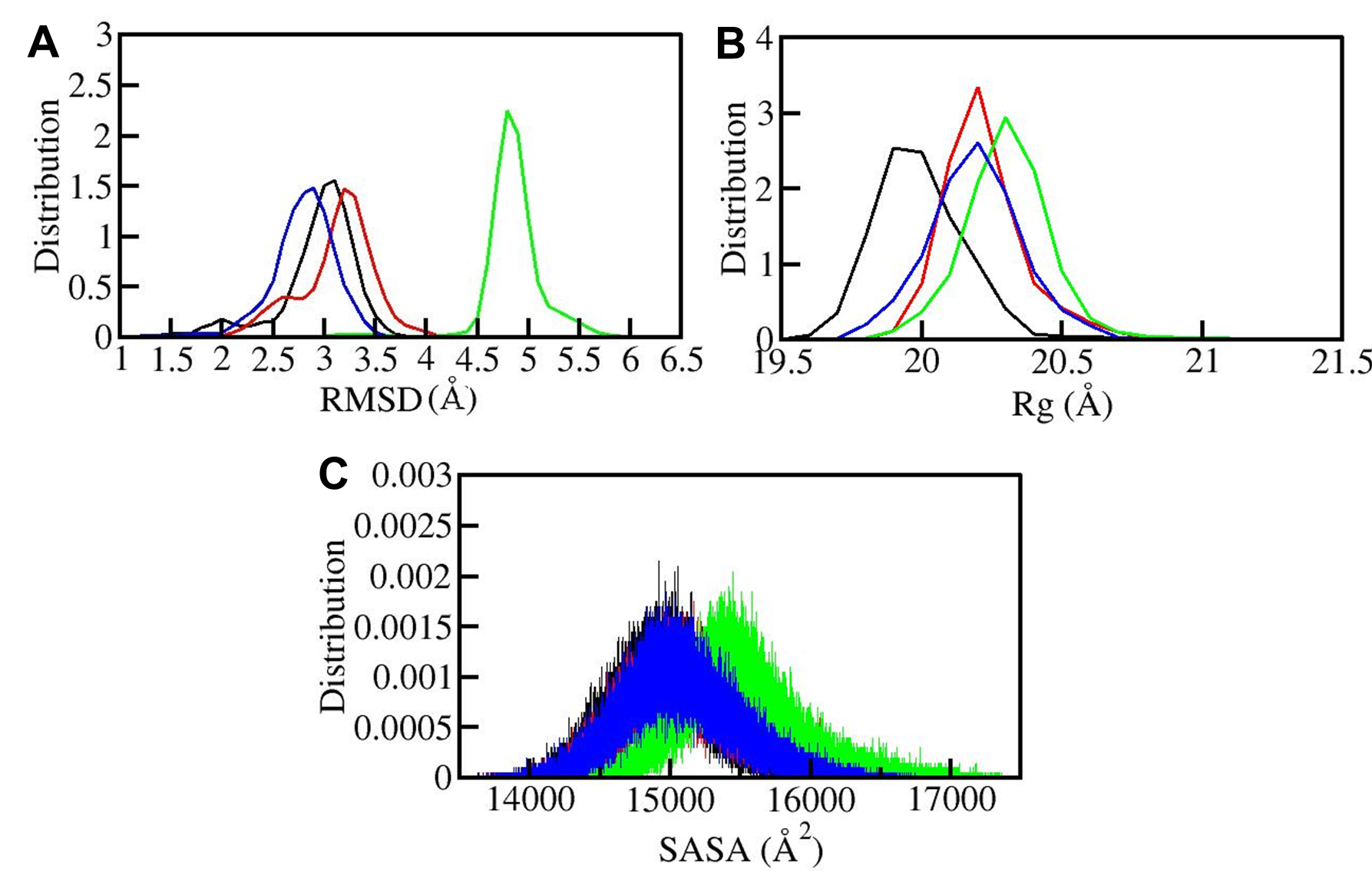
**

**Figure S2:** Probability distribution function plots for (A) RMSD, (B) *Rg*, and (C) SASA values during the simulation. Black, red, green, and blue represent values for WT, K127M, T256A, Y298A.

**Table S1.** Sequence-based prediction of mutations associated with SGK1.

| **S. No.** | **Mutation** | **PolyPhen2** | **Prediction** | **Provean Score** | **Prediction** | **SIFT Score** | **Prediction** | **FATHMM Prediction** | **FATHMM Score** |
| --- | --- | --- | --- | --- | --- | --- | --- | --- | --- |
|  | Q82R | 0 | Benign | -0.75 | Neutral | 0.099 | Tolerated | Tolerated | -0.9 |
|  | Q82E | 0.001 | Benign | -0.23 | Neutral | 0.873 | Tolerated | Tolerated | -0.23 |
|  | I83V | 0.003 | Benign | -0.47 | Neutral | 0.347 | Tolerated | Tolerated | -0.26 |
|  | N84S | 0.048 | Benign | -3.43 | Deleterious | 0.129 | Tolerated | Tolerated | -0.34 |
|  | L85F | 0.993 | Probably Damaging | -3.41 | Deleterious | 0.075 | Tolerated | Tolerated | -2.23 |
|  | P87L | 0.218 | Benign | -6.59 | Deleterious | 0.093 | Tolerated | Tolerated | -2.23 |
|  | P87R | 0.984 | Probably Damaging | -5.58 | Deleterious | 0.012 | Damaging | Tolerated | -1.63 |
|  | P87S | 0.283 | Benign | -4.67 | Deleterious | 0.053 | Tolerated | Tolerated | -1.28 |
|  | S88L | 0.999 | Probably Damaging | -4.68 | Deleterious | 0.003 | Damaging | Tolerated | -2.02 |
|  | S89T | 0.039 | Benign | -1.22 | Neutral | 0.279 | Tolerated | Tolerated | -1.17 |
|  | N90S | 0.066 | Benign | -2.71 | Deleterious | 0.105 | Tolerated | Tolerated | -0.49 |
|  | P91L | 0.774 | Possibly Damaging | -5.66 | Deleterious | 0.009 | Damaging | Tolerated | -2.11 |
|  | P91S | 0.147 | Benign | -4.1 | Deleterious | 0.107 | Tolerated | Tolerated | -1.14 |
|  | P91A | 0.218 | Benign | -4.41 | Deleterious | 0.099 | Tolerated | Tolerated | -1.32 |
|  | H92P | 0.109 | Benign | -3.46 | Deleterious | 0.084 | Tolerated | Tolerated | -2.04 |
|  | H92Y | 0.184 | Benign | -2.58 | Deleterious | 0.011 | Damaging | Tolerated | -2.16 |
|  | P95L | 0.997 | Probably Damaging | -8.77 | Deleterious | 0.124 | Tolerated | Tolerated | -0.5 |
|  | H99N | 0 | Benign | 0.07 | Neutral | 0.024 | Damaging | Tolerated | 0.45 |
|  | F100Y | 0.002 | Benign | -1.85 | Neutral | 0.498 | Tolerated | Tolerated | -1.94 |
|  | V103M | 0.911 | Possibly Damaging | -2.73 | Deleterious | 0.015 | Damaging | Damaging | -3.08 |
|  | L113V | 0.079 | Benign | -2.26 | Neutral | 0.06 | Tolerated | Tolerated | -0.21 |
|  | A115V | 0.987 | Probably Damaging | -3.84 | Deleterious | 0.674 | Tolerated | Tolerated | -1.26 |
|  | H117Q | 0.973 | Probably Damaging | -4.87 | Deleterious | 0.042 | Damaging | Tolerated | -1.95 |
|  | E120K | 0.009 | Benign | -2.61 | Deleterious | 0.133 | Tolerated | Tolerated | -0.44 |
|  | V122M | 0 | Benign | 0.7 | Neutral | 0.082 | Tolerated | Tolerated | -0.8 |
|  | Y124C | 1 | Probably Damaging | -8.53 | Deleterious | 0 | Damaging | Tolerated | -2.65 |
|  | K127M | 1 | Probably Damaging | -5.86 | Deleterious | 0 | Damaging | Damaging | -10.21 |
|  | Q130K | 0.085 | Benign | -2.89 | Deleterious | 0.52 | Tolerated | Tolerated | 1.27 |
|  | A133V | 0.02 | Benign | -0.78 | Neutral | 1 | Tolerated | Tolerated | -0.67 |
|  | A133T | 0.022 | Benign | -1.45 | Neutral | 0.265 | Tolerated | Tolerated | 0.14 |
|  | I134V | 0.067 | Benign | -0.72 | Neutral | 0.103 | Tolerated | Tolerated | -0.65 |
|  | K138R | 0.238 | Benign | -1.73 | Neutral | 0.03 | Damaging | Tolerated | -1.13 |
|  | K138Q | 0.985 | Probably Damaging | -2.31 | Neutral | 0.056 | Tolerated | Tolerated | -1.14 |
|  | K141M | 1 | Probably Damaging | -4.66 | Deleterious | 0.01 | Damaging | Damaging | -3.95 |
|  | H142R | 0.999 | Probably Damaging | -7 | Deleterious | 0 | Damaging | Tolerated | 0.03 |
|  | I143V | 0.715 | Possibly Damaging | -0.86 | Neutral | 0.22 | Tolerated | Tolerated | -0.43 |
|  | S145L | 0.615 | Possibly Damaging | -2.29 | Neutral | 0.002 | Damaging | Tolerated | -1.33 |
|  | R147Q | 1 | Probably Damaging | -3.65 | Deleterious | 0.003 | Damaging | Tolerated | 0.28 |
|  | R147W | 1 | Probably Damaging | -7.32 | Deleterious | 0 | Damaging | Tolerated | -1.82 |
|  | K155E | 0.02 | Benign | -2.43 | Neutral | 0.028 | Damaging | Tolerated | -0.79 |
|  | H156N | 1 | Probably Damaging | -6.62 | Deleterious | 0.074 | Tolerated | Damaging | -5.75 |
|  | P157L | 1 | Probably Damaging | -9.6 | Deleterious | 0 | Damaging | Damaging | -5.01 |
|  | P157A | 0.999 | Probably Damaging | -7.68 | Deleterious | 0 | Damaging | Damaging | -3.89 |
|  | F158L | 1 | Probably Damaging | -5.76 | Deleterious | 0 | Damaging | Tolerated | -2.19 |
|  | H163R | 0.153 | Benign | -6.59 | Deleterious | 0.174 | Tolerated | Tolerated | -1.21 |
|  | H163Y | 0.202 | Benign | -5.27 | Deleterious | 0.023 | Damaging | Tolerated | 0.69 |
|  | Q167R | 1 | Probably Damaging | -3.81 | Deleterious | 0 | Damaging | Tolerated | -1.65 |
|  | D170E | 0 | Benign | -1.14 | Neutral | 0.495 | Tolerated | Tolerated | -0.44 |
|  | L172W | 1 | Probably Damaging | -5.7 | Deleterious | 0 | Damaging | Damaging | -5.31 |
|  | G181V | 1 | Probably Damaging | -8.54 | Deleterious | 0 | Damaging | Damaging | -5.51 |
|  | Y186F | 0 | Benign | 0.72 | Neutral | 1 | Tolerated | Tolerated | -0.22 |
|  | Y186C | 0.996 | Probably Damaging | -6.04 | Deleterious | 0.001 | Damaging | Tolerated | -2.04 |
|  | R192H | 0.897 | Possibly Damaging | -4.26 | Deleterious | 0.022 | Damaging | Tolerated | -2.56 |
|  | R192C | 1 | Probably Damaging | -6.86 | Deleterious | 0 | Damaging | Damaging | -5.73 |
|  | C193Y | 0.002 | Benign | -1.03 | Neutral | 1 | Tolerated | Tolerated | -0.75 |
|  | C193R | 0.001 | Benign | -1.54 | Neutral | 0.615 | Tolerated | Tolerated | 1.19 |
|  | P197L | 0.152 | Benign | -5.48 | Deleterious | 0.115 | Tolerated | Tolerated | -2.59 |
|  | P197R | 0.99 | Probably Damaging | -4.71 | Deleterious | 0.088 | Tolerated | Tolerated | -1.62 |
|  | R198P | 1 | Probably Damaging | -6.77 | Deleterious | 0 | Damaging | Damaging | -3.28 |
|  | R198Q | 1 | Probably Damaging | -3.87 | Deleterious | 0 | Damaging | Tolerated | -0.16 |
|  | R200H | 1 | Probably Damaging | -4.15 | Deleterious | 0.001 | Damaging | Damaging | -4.46 |
|  | R200C | 1 | Probably Damaging | -6.73 | Deleterious | 0.004 | Damaging | Damaging | -4.55 |
|  | Y202C | 1 | Probably Damaging | -8.71 | Deleterious | 0 | Damaging | Damaging | -4.07 |
|  | A207V | 0.979 | Probably Damaging | -3.24 | Deleterious | 0.551 | Tolerated | Tolerated | -0.82 |
|  | S208I | 1 | Probably Damaging | -5.09 | Deleterious | 0.002 | Damaging | Damaging | -3.29 |
|  | G211S | 0.999 | Probably Damaging | -5.25 | Deleterious | 0.033 | Damaging | Tolerated | 0.75 |
|  | Y212H | 0.954 | Possibly Damaging | -4.81 | Deleterious | 0.11 | Tolerated | Tolerated | -2.61 |
|  | H214L | 0.999 | Probably Damaging | -10.57 | Deleterious | 0 | Damaging | Damaging | -6.96 |
|  | L216M | 0.567 | Possibly Damaging | -1.13 | Neutral | 0.094 | Tolerated | Tolerated | -1.65 |
|  | V219I | 0.177 | Benign | -0.22 | Neutral | 0.558 | Tolerated | Tolerated | 0.03 |
|  | L223V | 0.915 | Possibly Damaging | -2.87 | Deleterious | 0.014 | Damaging | Tolerated | -2.7 |
|  | L230P | 1 | Probably Damaging | -6.76 | Deleterious | 0 | Damaging | Damaging | -7.06 |
|  | D231E | 0.995 | Probably Damaging | -3.85 | Deleterious | 0.009 | Damaging | Tolerated | -2.74 |
|  | S232A | 0.005 | Benign | -1.52 | Neutral | 0.292 | Tolerated | Tolerated | -0.74 |
|  | S232T | 0.384 | Benign | -1.95 | Neutral | 0.136 | Tolerated | Tolerated | -0.96 |
|  | I236V | 0.009 | Benign | -0.21 | Neutral | 0.472 | Tolerated | Tolerated | 0.21 |
|  | L238F | 1 | Probably Damaging | -3.84 | Deleterious | 0.001 | Damaging | Damaging | -5.2 |
|  | N247K | 0.786 | Possibly Damaging | -2.6 | Deleterious | 0.006 | Damaging | Tolerated | 1.19 |
|  | N251K | 0.01 | Benign | -1.19 | Neutral | 0.138 | Tolerated | Tolerated | -0.95 |
|  | S252N | 0 | Benign | 0.05 | Neutral | 0.132 | Tolerated | Tolerated | -1.55 |
|  | S252G | 0 | Benign | 1.46 | Neutral | 1 | Tolerated | Tolerated | -0.64 |
|  | S255C | 0.01 | Benign | -3.45 | Deleterious | 0.032 | Damaging | Damaging | -4.44 |
|  | S255A | 0 | Benign | -1.93 | Neutral | 0.117 | Tolerated | Tolerated | -1.36 |
|  | T256A | 0.995 | Probably Damaging | -4.8 | Deleterious | 0 | Damaging | Tolerated | -2.51 |
|  | T256D | 0.999 | Probably Damaging | -5.76 | Deleterious | 0 | Damaging | Tolerated | -2.81 |
|  | T256E | 1 | Probably Damaging | -5.76 | Deleterious | 0 | Damaging | Tolerated | -2.28 |
|  | P261L | 1 | Probably Damaging | -9.43 | Deleterious | 0 | Damaging | Tolerated | -2.28 |
|  | A265T | 1 | Probably Damaging | -3.48 | Deleterious | 0 | Damaging | Damaging | -6.68 |
|  | V268L | 0.995 | Probably Damaging | -2.39 | Neutral | 0.08 | Tolerated | Tolerated | -1.34 |
|  | V268M | 1 | Probably Damaging | -2.5 | Deleterious | 0 | Damaging | Tolerated | -2.95 |
|  | H270N | 0.004 | Benign | -0.67 | Neutral | 0.12 | Tolerated | Tolerated | 1.03 |
|  | P273S | 0.374 | Benign | -2.83 | Deleterious | 0.063 | Tolerated | Tolerated | -1.2 |
|  | R276K | 0.279 | Benign | -1.53 | Neutral | 0.213 | Tolerated | Tolerated | 0.2 |
|  | V278M | 1 | Probably Damaging | -2.61 | Deleterious | 0 | Damaging | Damaging | -4.19 |
|  | V286I | 1 | Probably Damaging | -0.88 | Neutral | 0.002 | Damaging | Tolerated | 0.54 |
|  | M290I | 1 | Probably Damaging | -3.54 | Deleterious | 0.001 | Damaging | Tolerated | -1.7 |
|  | G293S | 0.996 | Probably Damaging | -4.76 | Deleterious | 0.021 | Damaging | Damaging | -4.94 |
|  | P296R | 1 | Probably Damaging | -8.21 | Deleterious | 0 | Damaging | Damaging | -7.35 |
|  | Y298A | 1 | Probably Damaging | -8.44 | Deleterious | 0 | Damaging | Tolerated | 0.59 |
|  | R300Q | 0.993 | Probably Damaging | -3.17 | Deleterious | 0.398 | Tolerated | Tolerated | -0.58 |
|  | R300G | 0.999 | Probably Damaging | -6.05 | Deleterious | 0.001 | Damaging | Tolerated | -1.59 |
|  | T302I | 0.457 | Possibly Damaging | -2.9 | Deleterious | 0.457 | Tolerated | Tolerated | -1.4 |
|  | A303V | 0.76 | Possibly Damaging | -2.99 | Deleterious | 0.222 | Tolerated | Tolerated | -1.34 |
|  | A303T | 0.812 | Possibly Damaging | -2.41 | Neutral | 0.415 | Tolerated | Tolerated | -1.3 |
|  | D307E | 0.001 | Benign | -2.72 | Deleterious | 0.866 | Tolerated | Tolerated | 0.52 |
|  | D307Y | 0.811 | Possibly Damaging | -7.04 | Deleterious | 0.001 | Damaging | Tolerated | -2.84 |
|  | D307N | 0.05 | Benign | -2.78 | Deleterious | 0.174 | Tolerated | Tolerated | -0.83 |
|  | N308K | 0.994 | Probably Damaging | -4.28 | Deleterious | 1 | Tolerated | Tolerated | 0.37 |
|  | N308S | 0.931 | Possibly Damaging | -3.5 | Deleterious | 0.127 | Tolerated | Tolerated | -1.42 |
|  | I309S | 1 | Probably Damaging | -5.64 | Deleterious | 0 | Damaging | Damaging | -5.11 |
|  | I309F | 1 | Probably Damaging | -3.78 | Deleterious | 0 | Damaging | Damaging | -4.82 |
|  | L310R | 1 | Probably Damaging | -5.67 | Deleterious | 0 | Damaging | Tolerated | -2.58 |
|  | L310Q | 1 | Probably Damaging | -5.66 | Deleterious | 0 | Damaging | Damaging | -3.04 |
|  | N311K | 0.784 | Possibly Damaging | -2.77 | Deleterious | 0.068 | Tolerated | Tolerated | -0.5 |
|  | P318A | 0.007 | Benign | -3.14 | Deleterious | 0.263 | Tolerated | Tolerated | -2.18 |
|  | N319S | 0.005 | Benign | -2.26 | Neutral | 0.3 | Tolerated | Tolerated | -0.32 |
|  | I320N | 0.991 | Probably Damaging | -4.26 | Deleterious | 0.002 | Damaging | Damaging | -4.1 |
|  | I320V | 0.002 | Benign | 0.3 | Neutral | 0.704 | Tolerated | Tolerated | -0.07 |
|  | T321A | 0.008 | Benign | -1.19 | Neutral | 0.027 | Damaging | Tolerated | -0.31 |
|  | S323C | 0.869 | Possibly Damaging | -2.7 | Deleterious | 0.027 | Damaging | Tolerated | -2.85 |
|  | A324T | 0.999 | Probably Damaging | -2.97 | Deleterious | 0.008 | Damaging | Damaging | -4.18 |
|  | G330D | 0.957 | Probably Damaging | -4.56 | Deleterious | 0.004 | Damaging | Damaging | -6.23 |
|  | K334N | 1 | Probably Damaging | -4.53 | Deleterious | 0 | Damaging | Damaging | -3.3 |
|  | D335V | 0.961 | Probably Damaging | -7.47 | Deleterious | 0.01 | Damaging | Damaging | -6.11 |
|  | K338E | 0.107 | Benign | -2.03 | Neutral | 0.069 | Tolerated | Tolerated | 4.16 |
|  | R339Q | 1 | Probably Damaging | -3.78 | Deleterious | 0 | Damaging | Damaging | -8.04 |
|  | R339W | 1 | Probably Damaging | -7.5 | Deleterious | 0 | Damaging | Damaging | -12.3 |
|  | L340F | 0.998 | Probably Damaging | -3.65 | Deleterious | 0.001 | Damaging | Damaging | -4.85 |
|  | G341A | 1 | Probably Damaging | -5.64 | Deleterious | 0 | Damaging | Damaging | -5.15 |
|  | G341R | 1 | Probably Damaging | -7.46 | Deleterious | 0 | Damaging | Damaging | -6.75 |
|  | A342V | 0.07 | Benign | -2.36 | Neutral | 0.064 | Tolerated | Tolerated | -0.35 |
|  | K343Q | 0.02 | Benign | -1.35 | Neutral | 0.288 | Tolerated | Tolerated | -0.8 |
|  | D344Y | 0.764 | Possibly Damaging | -2.78 | Deleterious | 0.002 | Damaging | Tolerated | -0.86 |
|  | D345E | 1 | Probably Damaging | -3.48 | Deleterious | 0.001 | Damaging | Tolerated | 0.1 |
|  | D345N | 1 | Probably Damaging | -4.32 | Deleterious | 0.003 | Damaging | Tolerated | -0.24 |
|  | I349V | 0.069 | Benign | -0.68 | Neutral | 0.222 | Tolerated | Tolerated | -0.46 |
|  | I349L | 0.013 | Benign | -1.49 | Neutral | 0.083 | Tolerated | Tolerated | -2.44 |
|  | K350E | 0.829 | Possibly Damaging | -2.73 | Deleterious | 0.017 | Damaging | Tolerated | 0.11 |
|  | S351N | 0 | Benign | 1.91 | Neutral | 1 | Tolerated | Tolerated | -1.54 |
|  | V353G | 0.027 | Benign | -1.65 | Neutral | 0.158 | Tolerated | Tolerated | 3.98 |
|  | V353A | 0 | Benign | 0 | Neutral | 0.46 | Tolerated | Tolerated | 1.79 |
|  | V353I | 0 | Benign | -0.47 | Neutral | 0.271 | Tolerated | Tolerated | -0.94 |
|  | F354V | 1 | Probably Damaging | -6.62 | Deleterious | 0 | Damaging | Tolerated | -2.56 |
|  | N359Y | 0.998 | Probably Damaging | -6.9 | Deleterious | 0.001 | Damaging | Damaging | -4.01 |
|  | D362E | 0.003 | Benign | -2.74 | Deleterious | 0.069 | Tolerated | Tolerated | -0.73 |
|  | D362V | 0.809 | Possibly Damaging | -7.54 | Deleterious | 0.002 | Damaging | Tolerated | -1.55 |
|  | I364T | 0.016 | Benign | -0.94 | Neutral | 0.205 | Tolerated | Tolerated | 1.48 |
|  | N365K | 0.026 | Benign | -0.7 | Neutral | 0.443 | Tolerated | Tolerated | 0.92 |
|  | N365S | 0 | Benign | -0.42 | Neutral | 0.273 | Tolerated | Tolerated | 0.5 |
|  | I368T | 0.762 | Possibly Damaging | -4.09 | Deleterious | 0.001 | Damaging | Tolerated | -1.68 |
|  | T369A | 0 | Benign | -2.31 | Neutral | 0.375 | Tolerated | Tolerated | 0.38 |
|  | P371R | 1 | Probably Damaging | -8.5 | Deleterious | 0 | Damaging | Damaging | -3.42 |
|  | N373I | 0.005 | Benign | -5.15 | Deleterious | 0.194 | Tolerated | Tolerated | -1.94 |
|  | P374L | 1 | Probably Damaging | -9.46 | Deleterious | 0 | Damaging | Damaging | -4 |
|  | N375K | 0.009 | Benign | -3.29 | Deleterious | 0.237 | Tolerated | Tolerated | 1.46 |
|  | G378R | 1 | Probably Damaging | -6.88 | Deleterious | 0.001 | Damaging | Tolerated | -1.88 |

**Table S2**. Structure based prediction of mutations associated with SGK1.

| **S. No.** | **Mutation** | **mCSM ΔΔ*G*** | **mCSM Prediction** | **SDM2 ΔΔ*G*** | **SDM2 Prediction** | **MAESTRO ΔΔ*G*pred.** | **PremPS ΔΔ*G*** |
| --- | --- | --- | --- | --- | --- | --- | --- |
|  | Q82R | 0.135 | Stabilizing | 0 | Increased stability | 0.039199209 | -0.29 |
|  | Q82E | -0.317 | Destabilizing | 0 | Increased stability | 1.197647854 | 0.07 |
|  | I83V | -0.958 | Destabilizing | -2.31 | Reduced stability | 0.568223423 | 0.51 |
|  | N84S | -0.184 | Destabilizing | 0.33 | Increased stability | 1.151555729 | -0.06 |
|  | L85F | -1.446 | Destabilizing | -0.52 | Reduced stability | 0.384523145 | 0.49 |
|  | P87L | -0.422 | Destabilizing | 0.78 | Increased stability | 0.286875716 | 0.29 |
|  | P87R | -0.021 | Destabilizing | 0.43 | Increased stability | -0.094209474 | 0.29 |
|  | P87S | -0.534 | Destabilizing | 0.1 | Increased stability | 0.995805695 | 0.24 |
|  | S88L | -0.45 | Destabilizing | 0.58 | Increased stability | -0.498433702 | 0.48 |
|  | S89T | -0.663 | Destabilizing | 0.02 | Increased stability | 0.313306274 | 0.22 |
|  | N90S | -0.805 | Destabilizing | 0.16 | Increased stability | 0.368986474 | 0.53 |
|  | P91L | -0.281 | Destabilizing | 0.78 | Increased stability | 0.194813952 | 0.47 |
|  | P91S | -0.257 | Destabilizing | 0.1 | Increased stability | 0.951095762 | 0.34 |
|  | P91A | -0.344 | Destabilizing | 0.67 | Increased stability | 0.456728696 | 0.48 |
|  | H92P | 0.834 | Stabilizing | -1.57 | Reduced stability | 0.462523781 | 0.08 |
|  | H92Y | 0.983 | Stabilizing | -0.5 | Reduced stability | 0.926433706 | 0.06 |
|  | P95L | 0.767 | Stabilizing | 1.56 | Increased stability | -0.543175479 | 0.21 |
|  | H99N | -1.285 | Destabilizing | -0.79 | Reduced stability | 1.345369831 | 0.02 |
|  | F100Y | -0.128 | Destabilizing | 0.5 | Increased stability | 0.502812224 | 0.66 |
|  | V103M | -0.409 | Destabilizing | -0.94 | Reduced stability | -0.280171207 | 0.46 |
|  | L113V | -1.467 | Destabilizing | -1.45 | Reduced stability | -0.094343391 | 1.13 |
|  | A115V | -0.484 | Destabilizing | 0.57 | Increased stability | -1.610296701 | 0.07 |
|  | H117Q | -1.358 | Destabilizing | -0.47 | Reduced stability | 0.609710637 | 1.06 |
|  | E120K | -1.554 | Destabilizing | -0.49 | Reduced stability | 0.791684673 | 0.63 |
|  | V122M | -0.168 | Destabilizing | -0.6 | Reduced stability | 0.562532221 | -0.16 |
|  | Y124C | -1.636 | Destabilizing | -1 | Reduced stability | 0.023881526 | 1.9 |
|  | K127M | -1.668 | Destabilizing | 1.16 | Increased stability | -1.349978302 | 0.78 |
|  | Q130K | -0.832 | Destabilizing | -0.04 | Reduced stability | 0.48232769 | 0.48 |
|  | A133V | -0.558 | Destabilizing | -0.98 | Reduced stability | 0.728817051 | -0.39 |
|  | A133T | -1.429 | Destabilizing | -0.81 | Reduced stability | 0.947510999 | 0.49 |
|  | I134V | -1.353 | Destabilizing | -2.33 | Reduced stability | 0.731284891 | 0.82 |
|  | K138R | -0.174 | Destabilizing | 0.06 | Increased stability | 0.540410659 | 0.7 |
|  | K138Q | 0.026 | Stabilizing | 0.02 | Increased stability | 1.120984636 | 0.73 |
|  | K141M | -2.402 | Highly Destabilizing | 0.72 | Increased stability | -0.184838151 | 0.57 |
|  | H142R | -1.292 | Destabilizing | -0.82 | Reduced stability | 0.687993139 | 1.26 |
|  | I143V | -1.052 | Destabilizing | 0.02 | Increased stability | 0.456278827 | 0.85 |
|  | S145L | -1.34 | Destabilizing | 0.69 | Increased stability | -0.75700717 | 0.03 |
|  | R147Q | -0.821 | Destabilizing | -0.37 | Reduced stability | 1.267826606 | 0.92 |
|  | R147W | -0.831 | Destabilizing | 0.12 | Increased stability | 0.112428432 | 0.43 |
|  | K155E | -0.256 | Destabilizing | 0.17 | Increased stability | 1.157572787 | 0.68 |
|  | H156N | 0.277 | Stabilizing | -0.97 | Reduced stability | 1.467440076 | 0.32 |
|  | P157L | -0.559 | Destabilizing | 1.18 | Increased stability | 0.748989204 | 0.42 |
|  | P157A | -1.478 | Destabilizing | 1.21 | Increased stability | 0.655591264 | 1.1 |
|  | F158L | -1.81 | Destabilizing | -1.66 | Reduced stability | 1.304909103 | 1.79 |
|  | H163R | -0.931 | Destabilizing | -0.69 | Reduced stability | 0.269722362 | 0.92 |
|  | H163Y | 0.796 | Stabilizing | -0.47 | Reduced stability | 0.378152448 | -0.07 |
|  | Q167R | -0.408 | Destabilizing | -0.13 | Reduced stability | -0.020995188 | 0.75 |
|  | D170E | -0.395 | Destabilizing | 0.06 | Increased stability | 0.644408031 | 0.12 |
|  | L172W | -1.645 | Destabilizing | -1.28 | Reduced stability | -1.099350823 | 2.01 |
|  | G181V | -0.577 | Destabilizing | -0.43 | Reduced stability | -0.62560093 | 0.53 |
|  | Y186F | 0.277 | Stabilizing | -0.46 | Reduced stability | -0.054558349 | -0.73 |
|  | Y186C | -0.559 | Destabilizing | -0.28 | Reduced stability | -0.072833716 | 0.71 |
|  | R192H | -1.478 | Destabilizing | -0.67 | Reduced stability | 1.194721896 | 0.65 |
|  | R192C | -1.81 | Destabilizing | -0.84 | Reduced stability | 1.258241477 | 0.41 |
|  | C193Y | -0.931 | Destabilizing | 0.09 | Increased stability | -0.188540709 | 0.17 |
|  | C193R | 0.796 | Stabilizing | 0.08 | Increased stability | 0.535910203 | 0.36 |
|  | P197L | -0.408 | Destabilizing | 2.24 | Increased stability | -0.302482844 | 0.47 |
|  | P197R | -0.395 | Destabilizing | 1.83 | Increased stability | -0.155081145 | 0.96 |
|  | R198P | -1.645 | Destabilizing | -3.35 | Reduced stability | 2.102406637 | 1.1 |
|  | R198Q | -0.577 | Destabilizing | -1.1 | Reduced stability | 0.854492606 | 1.05 |
|  | R200H | -1.833 | Destabilizing | -1.48 | Reduced stability | 1.138002406 | 1.39 |
|  | R200C | -1.811 | Destabilizing | -2.15 | Reduced stability | 1.002160267 | 0.67 |
|  | Y202C | -1.432 | Destabilizing | -0.99 | Reduced stability | 1.883341335 | 2.75 |
|  | A207V | 0.963 | Stabilizing | -1.14 | Reduced stability | 0.86688236 | 0.46 |
|  | S208I | 0.379 | Stabilizing | 0.81 | Increased stability | -0.654033588 | 0.33 |
|  | G211S | -1.123 | Destabilizing | -1.51 | Reduced stability | 0.89836356 | 1.08 |
|  | Y212H | -0.031 | Destabilizing | -0.65 | Reduced stability | 0.576371242 | 0.38 |
|  | H214L | -0.725 | Destabilizing | 1.51 | Increased stability | 0.352632672 | 0.18 |
|  | L216M | -0.568 | Destabilizing | -0.26 | Reduced stability | -0.025151058 | 0.69 |
|  | V219I | -0.797 | Destabilizing | -0.47 | Reduced stability | -0.053859133 | -0.45 |
|  | L223V | -1.854 | Destabilizing | -2.78 | Reduced stability | 1.360088093 | 1.62 |
|  | L230P | -1.955 | Destabilizing | -4.32 | Reduced stability | 1.087887667 | 2.64 |
|  | D231E | -0.97 | Destabilizing | 0.07 | Increased stability | -0.446748659 | 0.72 |
|  | S232A | -0.19 | Destabilizing | 0.4 | Increased stability | 0.349305857 | 0.28 |
|  | S232T | -0.539 | Destabilizing | 0.22 | Increased stability | -0.014185201 | 0.53 |
|  | I236V | -1.431 | Destabilizing | -2.16 | Reduced stability | 0.05210941 | 0.39 |
|  | L238F | -1.57 | Destabilizing | -0.59 | Reduced stability | -0.259245511 | 0.77 |
|  | N247K | -0.412 | Destabilizing | -0.5 | Reduced stability | 1.804278009 | 0.92 |
|  | N251K | 0.104 | Stabilizing | 0.45 | Increased stability | 0.538120129 | 0.17 |
|  | S252N | -0.057 | Destabilizing | 0.22 | Increased stability | 0.233636815 | 0.1 |
|  | S252G | -0.165 | Destabilizing | 0.16 | Increased stability | 0.945360859 | -0.17 |
|  | S255C | -0.243 | Destabilizing | 0.37 | Increased stability | 0.721232799 | 0.68 |
|  | S255A | -0.408 | Destabilizing | 0.62 | Increased stability | 0.632275802 | 0.51 |
|  | T256A | -0.663 | Destabilizing | 0.12 | Increased stability | 1.122536392 | 0.98 |
|  | T256D | 0.311 | Stabilizing | 0.12 | Increased stability | 0.139757093 | 0.95 |
|  | T256E | 0.236 | Stabilizing | 0.18 | Increased stability | -0.003349059 | 0.95 |
|  | P261L | -0.321 | Destabilizing | -0.58 | Reduced stability | 0.633935937 | 0.46 |
|  | A265T | -1.29 | Destabilizing | -2.54 | Reduced stability | 0.03004454 | 1.38 |
|  | V268L | -0.574 | Destabilizing | 0.68 | Increased stability | 0.492249532 | 0.55 |
|  | V268M | -0.382 | Destabilizing | 0.26 | Increased stability | 0.646423847 | 0.89 |
|  | H270N | -0.259 | Destabilizing | -0.48 | Reduced stability | -0.983032628 | 0.37 |
|  | P273S | -0.406 | Destabilizing | -0.63 | Reduced stability | 0.856646797 | 0.92 |
|  | R276K | -1.366 | Destabilizing | -0.24 | Reduced stability | 0.325007775 | 0.45 |
|  | V278M | -1.397 | Destabilizing | -0.69 | Reduced stability | 1.294984031 | 1.18 |
|  | V286I | -0.301 | Destabilizing | -0.23 | Reduced stability | 0.671376433 | 0.47 |
|  | M290I | -0.33 | Destabilizing | -0.62 | Reduced stability | 1.23237051 | 0.86 |
|  | G293S | -1.125 | Destabilizing | -3.57 | Reduced stability | -0.02350835 | 0.72 |
|  | P296R | -1.562 | Destabilizing | -1.23 | Reduced stability | -0.007436253 | 1.21 |
|  | Y298A | -1.146 | Destabilizing | 0.68 | Increased stability | 1.598594251 | 0.75 |
|  | R300Q | -0.056 | Destabilizing | -0.06 | Reduced stability | 1.386783385 | 0.68 |
|  | R300G | -0.436 | Destabilizing | -0.35 | Reduced stability | 1.483183022 | 0.69 |
|  | T302I | -0.087 | Destabilizing | 0.78 | Increased stability | 0.891450906 | 0.16 |
|  | A303V | -0.701 | Destabilizing | -0.84 | Reduced stability | 0.207534052 | 0.62 |
|  | A303T | -1.035 | Destabilizing | -1.53 | Reduced stability | 0.217197749 | 0.81 |
|  | D307E | -0.798 | Destabilizing | 1.49 | Increased stability | -0.088186087 | 0.13 |
|  | D307Y | -0.53 | Destabilizing | 0.77 | Increased stability | -0.303118555 | 0.41 |
|  | D307N | -0.654 | Destabilizing | 0.02 | Increased stability | 0.602200182 | 0.13 |
|  | N308K | -0.339 | Destabilizing | 1.18 | Increased stability | 1.290401266 | 0.72 |
|  | N308S | -1.145 | Destabilizing | 0.11 | Increased stability | 1.10826124 | 0.73 |
|  | I309S | -3.247 | Highly Destabilizing | -1.84 | Reduced stability | 2.091932249 | 2.01 |
|  | I309F | -1.599 | Destabilizing | -0.82 | Reduced stability | 1.119189553 | 0.78 |
|  | L310R | -0.896 | Destabilizing | -0.29 | Reduced stability | 0.787087147 | 1.24 |
|  | L310Q | -1.491 | Destabilizing | -0.41 | Reduced stability | 0.668047222 | 1.4 |
|  | N311K | -0.04 | Destabilizing | 0.7 | Increased stability | 0.562735651 | 0.03 |
|  | P318A | -0.436 | Destabilizing | -0.36 | Reduced stability | 0.86164771 | 0.51 |
|  | N319S | -0.106 | Destabilizing | -1.46 | Reduced stability | 1.261975831 | -0.17 |
|  | I320N | -1.993 | Destabilizing | -2.93 | Reduced stability | 0.039945371 | 1.67 |
|  | I320V | -1.53 | Destabilizing | -2.46 | Reduced stability | 0.804680586 | 0.08 |
|  | T321A | -0.568 | Destabilizing | -0.08 | Reduced stability | 1.24783622 | 0.69 |
|  | S323C | -0.298 | Destabilizing | 0.49 | Increased stability | 0.217026792 | 0.38 |
|  | A324T | -1.715 | Destabilizing | -2.54 | Reduced stability | 1.149430189 | 1.2 |
|  | G330D | -2.615 | Highly Destabilizing | 0.32 | Increased stability | 0.601033645 | 1.25 |
|  | K334N | -0.881 | Destabilizing | -0.08 | Reduced stability | 1.114960692 | 1.18 |
|  | D335V | -0.082 | Destabilizing | 0.74 | Increased stability | 1.505271342 | 0.3 |
|  | K338E | 0.02 | Stabilizing | 0.17 | Increased stability | -0.106370704 | 0.47 |
|  | R339Q | -1.554 | Destabilizing | -2.4 | Reduced stability | 1.040070956 | 1.56 |
|  | R339W | -1.436 | Destabilizing | -0.44 | Reduced stability | 0.279978487 | 0.4 |
|  | L340F | -1.536 | Destabilizing | -0.38 | Reduced stability | 1.012990018 | 0.97 |
|  | G341A | -0.955 | Destabilizing | -1.89 | Reduced stability | 0.688539857 | 1.26 |
|  | G341R | -1.296 | Destabilizing | -3.66 | Reduced stability | 0.222998598 | 1.13 |
|  | A342V | -0.707 | Destabilizing | -0.12 | Reduced stability | 0.462076877 | 0.47 |
|  | K343Q | -0.075 | Destabilizing | 0.02 | Increased stability | 1.152941955 | 0.25 |
|  | D344Y | -0.265 | Destabilizing | -0.09 | Reduced stability | -0.050059805 | 0.2 |
|  | D345E | -0.763 | Destabilizing | -0.01 | Reduced stability | 0.934677734 | 0.72 |
|  | D345N | -1.772 | Destabilizing | 0.49 | Increased stability | 1.772347121 | 0.7 |
|  | I349V | -0.888 | Destabilizing | -2.56 | Reduced stability | 1.487619884 | 0.98 |
|  | I349L | -0.393 | Destabilizing | 0.36 | Increased stability | 1.11222283 | 0.9 |
|  | K350E | -1.13 | Destabilizing | 1.02 | Increased stability | 1.04502442 | 1.15 |
|  | S351N | -0.706 | Destabilizing | -0.17 | Reduced stability | -0.123859575 | -0.23 |
|  | V353G | -1.579 | Destabilizing | -0.72 | Reduced stability | 0.261148437 | 0.69 |
|  | V353A | -1.275 | Destabilizing | 0.84 | Increased stability | 0.028686107 | 0.05 |
|  | V353I | -0.553 | Destabilizing | 0.35 | Increased stability | -0.034254921 | 0.2 |
|  | F354V | -1.807 | Destabilizing | -2.5 | Reduced stability | 1.693649021 | 1.75 |
|  | N359Y | -0.375 | Destabilizing | 0.56 | Increased stability | 0.454692815 | 0.36 |
|  | D362E | -0.94 | Destabilizing | 0.84 | Increased stability | 1.237989446 | 0.7 |
|  | D362V | 0.196 | Stabilizing | 0.23 | Increased stability | 1.510257191 | 0.16 |
|  | I364T | -0.926 | Destabilizing | -1.5 | Reduced stability | -0.266636448 | 0.63 |
|  | N365K | -0.055 | Destabilizing | 0.01 | Increased stability | -0.154812598 | 0.11 |
|  | N365S | -0.27 | Destabilizing | -0.56 | Reduced stability | -0.104571315 | -0.05 |
|  | I368T | -1.919 | Destabilizing | -0.89 | Reduced stability | 1.030669574 | 1.42 |
|  | T369A | -0.522 | Destabilizing | 0.81 | Increased stability | 0.916208836 | 0.39 |
|  | P371R | -0.737 | Destabilizing | 0.43 | Increased stability | 0.563165854 | 1.01 |
|  | N373I | -0.171 | Destabilizing | 1.26 | Increased stability | 0.433811517 | -0.06 |
|  | P374L | -0.947 | Destabilizing | -0.78 | Reduced stability | -0.245139454 | 0.35 |
|  | N375K | 0.064 | Stabilizing | -0.54 | Reduced stability | 0.267097027 | 0.09 |
|  | G378R | -0.383 | Destabilizing | 0 | Increased stability | -0.075309019 | 0.34 |

**Table S3**. Pathogenicity prediction of mutations associated with SGK1.

| **S. No.** | **Mutation** | **SNPs&GO** | **SNPs&GO** | **PON-P2** | **PON-P2** | **Pmut** | **Pmut** |
| --- | --- | --- | --- | --- | --- | --- | --- |
|  | Q82E | 0.023 | Neutral | 0.73 | Unknown | 0.15 (94%) | Neutral |
|  | I83V | 0.015 | Neutral | 0.645 | Unknown | 0.18 (93%) | Neutral |
|  | L85F | 0.226 | Neutral | 0.573 | Unknown | 0.42 (85%) | Neutral |
|  | P87L | 0.49 | Neutral | 0.83 | Pathogenic | 0.52 (79%) | Disease |
|  | P87R | 0.539 | Disease | 0.926 | Pathogenic | 0.50 (79%) | Disease |
|  | P87S | 0.445 | Neutral | 0.807 | Pathogenic | 0.40 (86%) | Neutral |
|  | S88L | 0.389 | Neutral | 0.769 | Unknown | 0.41 (86%) | Neutral |
|  | S89T | 0.053 | Neutral | 0.579 | Unknown | 0.11 (95%) | Neutral |
|  | N90S | 0.118 | Neutral | 0.32 | Unknown | 0.10 (96%) | Neutral |
|  | P91L | 0.097 | Neutral | 0.622 | Unknown | 0.45 (84%) | Neutral |
|  | P91S | 0.305 | Neutral | 0.417 | Unknown | 0.36 (87%) | Neutral |
|  | P91A | 0.179 | Neutral | 0.351 | Unknown | 0.34 (88%) | Neutral |
|  | H92P | 0.381 | Neutral | 0.697 | Unknown | 0.18 (93%) | Neutral |
|  | H92Y | 0.218 | Neutral | 0.382 | Unknown | 0.52 (79%) | Disease |
|  | H99N | 0.066 | Neutral | 0.559 | Unknown | 0.06 (97%) | Neutral |
|  | F100Y | 0.126 | Neutral | 0.572 | Unknown | 0.12 (95%) | Neutral |
|  | V103M | 0.272 | Neutral | 0.668 | Unknown | 0.16 (94%) | Neutral |
|  | L113V | 0.2 | Neutral | 0.727 | Unknown | 0.13 (95%) | Neutral |
|  | H117Q | 0.214 | Neutral | 0.816 | Unknown | 0.29 (90%) | Neutral |
|  | E120K | 0.164 | Neutral | 0.551 | Unknown | 0.14 (94%) | Neutral |
|  | V122M | 0.056 | Neutral | 0.477 | Unknown | 0.05 (97%) | Neutral |
|  | Y124C | 0.655 | Disease | 0.873 | Pathogenic | 0.31 (89%) | Neutral |
|  | K127M | 0.579 | Disease | 0.836 | Pathogenic | 0.72 (86%) | Disease |
|  | Q130K | 0.068 | Neutral | 0.619 | Unknown | 0.04 (98%) | Neutral |
|  | A133V | 0.044 | Neutral | 0.8 | Pathogenic | 0.06 (97%) | Neutral |
|  | A133T | 0.029 | Neutral | 0.729 | Unknown | 0.06 (97%) | Neutral |
|  | I134V | 0.04 | Neutral | 0.523 | Unknown | 0.08 (96%) | Neutral |
|  | K138R | 0.113 | Neutral | 0.684 | Unknown | 0.13 (95%) | Neutral |
|  | K141M | 0.177 | Neutral | 0.739 | Unknown | 0.11 (95%) | Neutral |
|  | H142R | 0.771 | Disease | 0.837 | Pathogenic | 0.08 (96%) | Neutral |
|  | I143V | 0.035 | Neutral | 0.739 | Unknown | 0.04 (98%) | Neutral |
|  | R147Q | 0.657 | Disease | 0.714 | Unknown | 0.55 (81%) | Disease |
|  | R147W | 0.672 | Disease | 0.864 | Pathogenic | 0.77 (88%) | Disease |
|  | K155E | 0.442 | Neutral | 0.841 | Pathogenic | 0.08 (96%) | Neutral |
|  | H156N | 0.624 | Disease | 0.58 | Unknown | 0.16 (94%) | Neutral |
|  | P157L | 0.646 | Disease | 0.692 | Unknown | 0.72 (86%) | Disease |
|  | P157A | 0.735 | Disease | 0.392 | Unknown | 0.57 (81%) | Disease |
|  | F158L | 0.657 | Disease | 0.407 | Unknown | 0.72 (86%) | Disease |
|  | H163R | 0.14 | Neutral | 0.631 | Unknown | 0.46 (84%) | Neutral |
|  | Q167R | 0.819 | Disease | 0.61 | Unknown | 0.77 (88%) | Disease |
|  | D170E | 0.038 | Neutral | 0.529 | Unknown | 0.07 (97%) | Neutral |
|  | L172W | 0.701 | Disease | 0.819 | Pathogenic | 0.79 (89%) | Disease |
|  | G181V | 0.836 | Disease | 0.928 | Pathogenic | 0.72 (86%) | Disease |
|  | Y186C | 0.538 | Disease | 0.871 | Pathogenic | 0.79 (89%) | Disease |
|  | R192H | 0.666 | Disease | 0.654 | Unknown | 0.10 (95%) | Neutral |
|  | R192C | 0.275 | Neutral | 0.606 | Unknown | 0.32 (89%) | Neutral |
|  | R198P | 0.87 | Disease | 0.89 | Pathogenic | 0.79 (89%) | Disease |
|  | R198Q | 0.514 | Disease | 0.754 | Unknown | 0.07 (97%) | Neutral |
|  | R200H | 0.778 | Disease | 0.656 | Unknown | 0.79 (89%) | Disease |
|  | R200C | 0.635 | Disease | 0.886 | Pathogenic | 0.08 (96%) | Neutral |
|  | Y202C | 0.79 | Disease | 0.552 | Unknown | 0.72 (86%) | Disease |
|  | A207V | 0.498 | Neutral | 0.587 | Unknown | 0.04 (98%) | Neutral |
|  | S208I | 0.726 | Disease | 0.761 | Pathogenic | 0.04 (98%) | Neutral |
|  | G211S | 0.397 | Neutral | 0.612 | Unknown | 0.07 (97%) | Neutral |
|  | Y212H | 0.568 | Disease | 0.785 | Pathogenic | 0.05 (97%) | Neutral |
|  | H214L | 0.813 | Disease | 0.52 | Unknown | 0.72 (86%) | Disease |
|  | L216M | 0.082 | Neutral | 0.443 | Unknown | 0.07 (97%) | Neutral |
|  | L223V | 0.535 | Disease | 0.573 | Unknown | 0.54 (80%) | Disease |
|  | L230P | 0.789 | Disease | 0.943 | Pathogenic | 0.79 (89%) | Disease |
|  | D231E | 0.644 | Disease | 0.798 | Unknown | 0.66 (85%) | Disease |
|  | S232A | 0.068 | Neutral | 0.46 | Unknown | 0.03 (98%) | Neutral |
|  | I236V | 0.028 | Neutral | 0.717 | Unknown | 0.10 (96%) | Neutral |
|  | L238F | 0.532 | Disease | 0.567 | Unknown | 0.77 (88%) | Disease |
|  | N247K | 0.1 | Neutral | 0.665 | Unknown | 0.74 (87%) | Disease |
|  | S252N | 0.03 | Neutral | 0.619 | Unknown | 0.06 (97%) | Neutral |
|  | S255C | 0.057 | Neutral | 0.866 | Pathogenic | 0.12 (95%) | Neutral |
|  | S255A | 0.36 | Neutral | 0.677 | Unknown | 0.11 (95%) | Neutral |
|  | T256A | 0.577 | Disease | 0.881 | Pathogenic | 0.82 (90%) | Disease |
|  | T256D | 0.705 | Disease | 0.927 | Pathogenic | 0.82 (90%) | Disease |
|  | T256E | 0.765 | Disease | 0.882 | Pathogenic | 0.82 (90%) | Disease |
|  | P261L | 0.72 | Disease | 0.525 | Unknown | 0.56 (81%) | Disease |
|  | A265T | 0.759 | Disease | 0.569 | Unknown | 0.72 (86%) | Disease |
|  | V268L | 0.261 | Neutral | 0.388 | Unknown | 0.11 (95%) | Neutral |
|  | V268M | 0.301 | Neutral | 0.552 | Unknown | 0.11 (95%) | Neutral |
|  | H270N | 0.09 | Neutral | 0.643 | Unknown | 0.08 (96%) | Neutral |
|  | P273S | 0.473 | Neutral | 0.385 | Unknown | 0.08 (96%) | Neutral |
|  | R276K | 0.044 | Neutral | 0.616 | Unknown | 0.06 (97%) | Neutral |
|  | V278M | 0.646 | Disease | 0.784 | Pathogenic | 0.79 (89%) | Disease |
|  | V286I | 0.225 | Neutral | 0.383 | Unknown | 0.24 (91%) | Neutral |
|  | M290I | 0.762 | Disease | 0.471 | Unknown | 0.72 (86%) | Disease |
|  | G293S | 0.778 | Disease | 0.417 | Unknown | 0.59 (82%) | Disease |
|  | P296R | 0.765 | Disease | 0.918 | Pathogenic | 0.72 (86%) | Disease |
|  | Y298A | 0.649 | Disease | 0.802 | Pathogenic | 0.81 (89%) | Disease |
|  | R300Q | 0.267 | Neutral | 0.879 | Pathogenic | 0.04 (98%) | Neutral |
|  | R300G | 0.079 | Neutral | 0.882 | Pathogenic | 0.12 (95%) | Neutral |
|  | T302I | 0.097 | Neutral | 0.663 | Unknown | 0.08 (96%) | Neutral |
|  | A303V | 0.221 | Neutral | 0.59 | Unknown | 0.09 (96%) | Neutral |
|  | A303T | 0.287 | Neutral | 0.569 | Unknown | 0.09 (96%) | Neutral |
|  | D307Y | 0.231 | Neutral | 0.621 | Unknown | 0.43 (85%) | Neutral |
|  | D307N | 0.573 | Disease | 0.602 | Unknown | 0.09 (96%) | Neutral |
|  | N308K | 0.072 | Neutral | 0.557 | Unknown | 0.08 (96%) | Neutral |
|  | N308S | 0.092 | Neutral | 0.412 | Unknown | 0.08 (96%) | Neutral |
|  | I309S | 0.813 | Disease | 0.709 | Unknown | 0.72 (86%) | Disease |
|  | I309F | 0.857 | Disease | 0.571 | Unknown | 0.70 (86%) | Disease |
|  | L310R | 0.693 | Disease | 0.889 | Pathogenic | 0.06 (97%) | Neutral |
|  | L310Q | 0.755 | Disease | 0.756 | Pathogenic | 0.39 (86%) | Neutral |
|  | N311K | 0.098 | Neutral | 0.645 | Unknown | 0.11 (95%) | Neutral |
|  | P318A | 0.021 | Neutral | 0.578 | Unknown | 0.24 (91%) | Neutral |
|  | N319S | 0.045 | Neutral | 0.877 | Pathogenic | 0.23 (91%) | Neutral |
|  | I320N | 0.511 | Disease | 0.879 | Pathogenic | 0.80 (89%) | Disease |
|  | I320V | 0.019 | Neutral | 0.676 | Unknown | 0.08 (96%) | Neutral |
|  | T321A | 0.099 | Neutral | 0.418 | Unknown | 0.09 (96%) | Neutral |
|  | S323C | 0.193 | Neutral | 0.483 | Unknown | 0.75 (88%) | Disease |
|  | A324T | 0.442 | Neutral | 0.862 | Unknown | 0.38 (87%) | Neutral |
|  | G330D | 0.251 | Neutral | 0.908 | Pathogenic | 0.61 (83%) | Disease |
|  | K334N | 0.523 | Disease | 0.595 | Unknown | 0.58 (82%) | Disease |
|  | D335V | 0.559 | Disease | 0.889 | Pathogenic | 0.78 (88%) | Disease |
|  | R339Q | 0.772 | Disease | 0.775 | Unknown | 0.72 (86%) | Disease |
|  | R339W | 0.773 | Disease | 0.852 | Pathogenic | 0.72 (86%) | Disease |
|  | L340F | 0.377 | Neutral | 0.604 | Unknown | 0.16 (94%) | Neutral |
|  | G341A | 0.527 | Disease | 0.876 | Pathogenic | 0.70 (86%) | Disease |
|  | G341R | 0.748 | Disease | 0.813 | Unknown | 0.70 (86%) | Disease |
|  | A342V | 0.118 | Neutral | 0.469 | Unknown | 0.31 (89%) | Neutral |
|  | K343Q | 0.164 | Neutral | 0.48 | Unknown | 0.28 (90%) | Neutral |
|  | D344Y | 0.225 | Neutral | 0.631 | Unknown | 0.42 (85%) | Neutral |
|  | D345E | 0.482 | Neutral | 0.785 | Pathogenic | 0.62 (83%) | Disease |
|  | D345N | 0.554 | Disease | 0.909 | Pathogenic | 0.32 (89%) | Neutral |
|  | I349V | 0.327 | Neutral | 0.328 | Unknown | 0.14 (94%) | Neutral |
|  | I349L | 0.127 | Neutral | 0.408 | Unknown | 0.10 (95%) | Neutral |
|  | K350E | 0.58 | Disease | 0.588 | Unknown | 0.77 (88%) | Disease |
|  | V353G | 0.075 | Neutral | 0.472 | Unknown | 0.12 (95%) | Neutral |
|  | V353A | 0.323 | Neutral | 0.503 | Unknown | 0.08 (96%) | Neutral |
|  | F354V | 0.755 | Disease | 0.677 | Unknown | 0.79 (89%) | Disease |
|  | N359Y | 0.5 | Neutral | 0.759 | Unknown | 0.79 (89%) | Disease |
|  | D362E | 0.254 | Neutral | 0.307 | Unknown | 0.12 (95%) | Neutral |
|  | D362V | 0.56 | Disease | 0.643 | Unknown | 0.28 (90%) | Neutral |
|  | I364T | 0.086 | Neutral | 0.575 | Unknown | 0.31 (89%) | Neutral |
|  | I368T | 0.495 | Neutral | 0.685 | Unknown | 0.38 (87%) | Neutral |
|  | T369A | 0.066 | Neutral | 0.444 | Unknown | 0.12 (95%) | Neutral |
|  | P371R | 0.754 | Disease | 0.913 | Pathogenic | 0.70 (86%) | Disease |
|  | N373I | 0.06 | Neutral | 0.84 | Pathogenic | 0.08 (96%) | Neutral |
|  | P374L | 0.723 | Disease | 0.822 | Pathogenic | 0.70 (86%) | Disease |
|  | N375K | 0.049 | Neutral | 0.783 | Unknown | 0.07 (96%) | Neutral |
|  | G378R | 0.621 | Disease | 0.881 | Pathogenic | 0.12 (95%) | Neutral |
